# Absorption of Broadband Low‐Frequency Sound Beyond the Thermodynamics Limit: An Acoustic Resonator With Magnetic Bearing

**DOI:** 10.1002/advs.76584

**Published:** 2026-07-16

**Authors:** Ying Hu, Zhe Zhang, Bohua Huang, Xue Han, Hallam Bastin Kilcoyne, Lixi Huang

**Affiliations:** ^1^ Department of Mechanical Engineering The University of Hong Kong Hong Kong China

**Keywords:** broadband absorption, low‐frequency sound absorption, negative stiffness, noise control, sound absorption material

## Abstract

This study reports the first experimental realization of magnetic negative stiffness to achieve a significant net gain in broadband, low‐frequency sound absorption, beyond the thermodynamic limit posed by the relative incompressibility of cavity fluid at low frequencies that otherwise necessitates impractically large absorbers. Analysis based on experimental data demonstrates a fivefold amplification of the absorption bandwidth within the lowest two octaves of human hearing; theoretically, this could be further enhanced with improved structural design and fabrication accuracy. The device employs a magnetic bearing structure with its moving shaft constrained by a mechanical ball bearing, resolving the rotational magnetic instability that otherwise causes significant parasitic stiffness, an issue that has prevented previous designs from succeeding. From an energy perspective, the device creates a potential well where a small incident wave activates a large kinetic response for viscothermal absorption. The proposed magnetic device offers a highly practical and scalable solution for engineering applications.

## Graphical abstract

This work presents a magnetic‐bearing acoustic resonator that overcomes the thermodynamics limit of sound absorption. By introducing tunable negative stiffness, it achieves broadband low‐frequency absorption within a compact cavity, far exceeding the performance achievable by classic mass‐tuned designs. The first experimental demonstration confirms a significant bandwidth expansion, offering a transformative strategy for low‐frequency noise control.

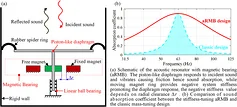

## 1 Introduction

Effective control of low‐frequency noise within compact spaces remains a formidable challenge in acoustics to this day. Because traditional noise control devices scale with acoustic wavelength, they are often impractically bulky for many applications. This limitation is particularly evident in the aerospace sector, where intense acoustic loads during rocket launch threaten structural integrity [1] and low‐frequency combustion instabilities jeopardize engine reliability [2]. Similar challenges arise in critical listening environments, where intrusive low‐frequency noise compromises sound quality [3]. Consequently, developing efficient, compact low‐frequency sound absorbers is essential for advancing both high‐performance engineering systems and architectural acoustics.

Due to their strong diffraction and deep penetration, low‐frequency sound waves render conventional acoustic barriers largely ineffective [4, 5]. Current mitigation strategies are primarily passive [6, 7, 8] and active [9, 10]. While active noise control (ANC) effectively cancels low‐frequency noise, its reliance on integrated sensors and actuators introduces high costs, system complexity, and environmental sensitivity. Conversely, passive methods, typically employing porous materials or resonator arrays, collectively described as acoustic metamaterials in the latest literature, are limited by the trade‐off between structural size and effective frequency band [11, 12, 13]. Although the use of a porous medium helps reduce cavity stiffness [14], the required cavity depth (e.g., 1/8 of the wavelength) remains physically impractical for frequencies in the range of 100 Hz [15, 16]. Existing passive approaches utilizing acoustic resonance (e.g., various Helmholtz resonator configurations) only achieve high absorption within narrow frequency bands when constrained to compact sizes. This limitation arises because compact devices inherently possess high cavity air stiffness. As a result, targeting low‐frequency resonance requires a significantly large acoustic mass, which produces sharp, narrow absorption peaks that fail to meet practical demands [17, 18]. This challenge is fundamentally governed by the high stiffness due to relative air incompressibility. Ideally, this issue is resolved by reducing system stiffness rather than increasing acoustic mass. However, low system stiffness typically necessitates deeper cavities and bulkier designs. Therefore, achieving a desirable bandwidth of sound absorption in the low‐frequency region requires harnessing negative stiffness. This paradigm decouples low‐frequency performance from compactness, directly addressing the decades‐long pursuit of broadband, compact low‐frequency absorbers, which remains the primary frontier in contemporary acoustics research.

Negative stiffness occurs when a structure, perturbed around its equilibrium position, generates a force in the same direction as the displacement, yielding a negative slope in the force‑displacement curve. Since magnetic attraction and repulsion forces scale with the inverse square of distance, magnetic negative stiffness presents a natural solution. Its potential for acoustic noise control in air was first theoretically explored in 2000 by Huang [19], who proposed a compact, reactive, side‐branch silencer with a multi‐piston system to counteract the positive stiffness of a compact air cavity. Subsequently, researchers began experimentally applying magnetic negative stiffness to acoustics. Zhao et al. [20] employed unilateral magnetic attraction (Figure 1a), which pre‐tensioned the membrane and excited higher‐order modes, ultimately increasing rather than decreasing the resonant frequency. Chiu et al. [21] balanced magnetic forces against cavity pressure to achieve a frequency reduction (Figure 1b), but the system proved highly sensitive and difficult to tune. Li et al. [22] adopted a symmetric magnetic configuration to cancel out the net force imbalance (Figure 1c); however, rotational instability of the iron component introduced membrane tension and dipole modes, undermining the intended piston‐like response. Previous attempts at negative stiffness have been hindered by two primary failure modes: initial force imbalance and rotational instability. Beyond magnetic designs, pneumatic‐buckling configurations [23] offer an alternative approach but risk exciting higher‐order modes. A critical distinction must be made: if the negative stiffness merely compensates for unavoidable structural positive stiffness, henceforth denoted as “parasitic stiffness”, without reducing the initial cavity air stiffness, the design's intended purpose remains unfulfilled. Accordingly, this study introduces a stiffness ratio κ to quantify the effective benefit of negative stiffness, defined as the ratio of the total change of cavity stiffness, which is the sum of the negative stiffness and parasitic stiffness, to the original stiffness of the pure air cavity, κ  = (*k*_neg_ + *k*_par_) /*k*_air_. When κ  =  0, the negative stiffness exactly cancels all parasitic positive stiffness, leaving the total system stiffness equal to the natural air‐cavity stiffness proportional to γ_air_ *P*_0_, where γ_air_ is the thermodynamic ratio of specific heats in cavity, and *P*_0_ is the static pressure which should be equal to the air pressure outside the cavity. When the original cavity stiffness is successfully and totally neutralized by magnetic force, we have κ  =   −1. Dynamic stability requires a positive residual stiffness, hence κ > −1. When the cavity is filled with porous material, sound propagation changes from an isentropic process to an isothermal process due to heat transfer between air particles and surfaces of pores. The effective fluid stiffness is reduced by a factor of γ_air_ =  7/5 for air dominated by diatomic molecules. The stiffness ratio is thus κ  =  1/γ_air_ −1  =   −2/7, determined by thermodynamics [14]. Note that the thermodynamics limit can also be improved from κ  =  0 if the fluid in the cavity is changed from air to polyatomic gases whose ratio of specific heats, denoted as γ_poly_, is closer to unity, and its isentropic bulk modulus becomes less than that of air of the same static pressure *P*_0_, giving κ  = γ_poly_ /γ_air_ − 1 < 0 with a lower limit of −2/7 also when γ_poly_ approaches unity. However, the use of special gas requires membrane sealing with potential engineering problem of molecular leakage through membrane and parasitic membrane tension. From time to time the use of porous material may not be preferred from hygienic and other considerations. We therefore consider the dual value of κ  =  0 for pure air cavity and κ  =   −2/7 for air cavity filled by porous materials or other substance to be the thermodynamics limit. As further analyzed in the Discussion section, experimental data fitting suggests that previous negative stiffness devices [22, 23] have yet to realize effective negative stiffness, −1 < κ < 0. While the proof of concept remains promising, achieving the intended performance requires great effort.

**FIGURE 1:**
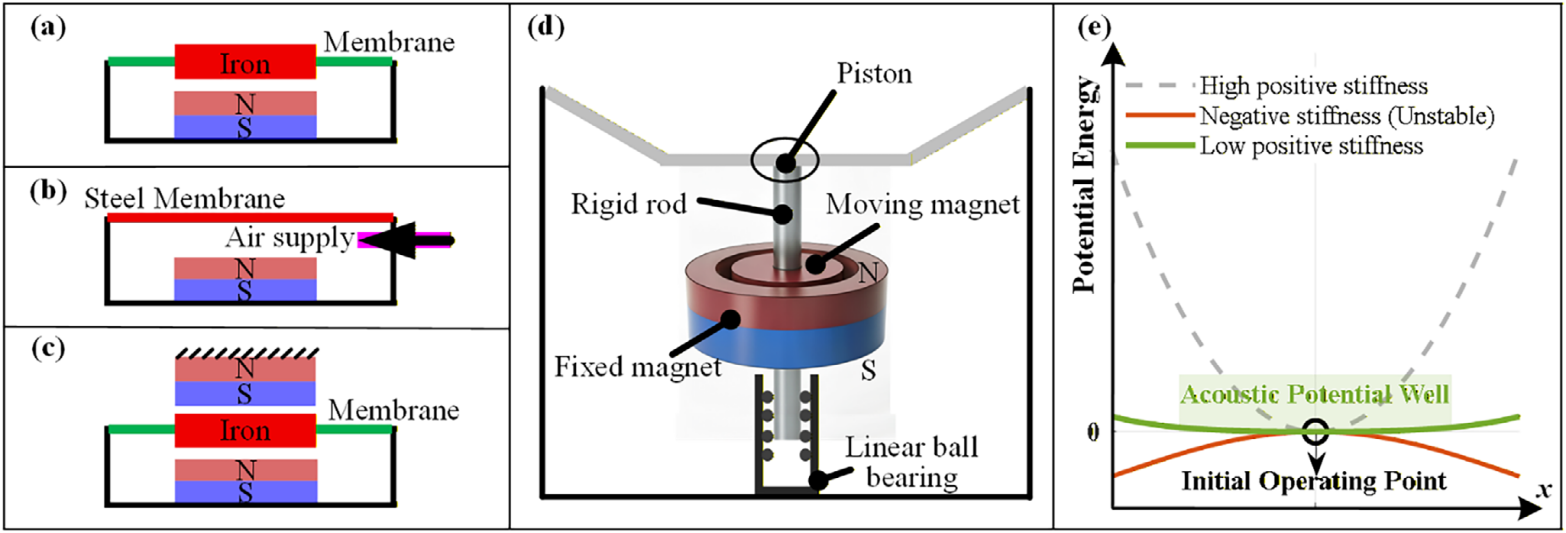
Schematic diagram of magnetic negative stiffness devices and acoustic potential well. (a) Unilateral magnet structure [20]; (b) Unilateral magnet with cavity pressure balance [21]; (c) Symmetrical magnet structure [22]; (d) The negative‐stiffness device introduced in this study, consisting of a fixed ring of outer magnet and a moving circular inner magnetic disk attached to a rigid rod inserted into a linear ball bearing. (e) Conceptual illustration of the acoustic potential well.

Figure 1d illustrates the proposed device, hereafter referred to as the “Acoustic Resonator with Magnetic Bearing” (aRMB), designed to stably harness negative magnetic stiffness. The configuration consists of horizontally aligned circular magnets with zero static force between them: the outer ring is fixed, while the inner disk attaches to a diaphragm, allowing axial motion under acoustic disturbance. Within a specific axial range, the disk experiences negative stiffness that partially offsets the positive cavity air stiffness. However, Earnshaw's theorem [24] dictates that permanent magnets alone cannot achieve stable static equilibrium in all degrees of freedom. To address the resulting angular instability (like that in Figure 1c), linear ball‐bearing houses a rigid rod attached to the moving disk, constraining angular motion while ensuring axial movement with low‐friction. The system inherently maintains radial stability (see Figure 3d), as the inner disk returns to a centrally aligned position following radial disturbances, allowing the magnetic pair to function as a contactless bearing. In contrast to the attraction‐based, face‐to‐face configuration in Figure 1c, the aRMB in Figure 1d utilizes repulsive forces between magnetic rings separated by a fixed radial air gap. This architecture allows a reduced radial gap within a practical range to significantly enhance negative stiffness, while the system operates at maximum stiffness at equilibrium without parasitic tension or competing positive stiffness effects. Calculations indicate that this magnetic bearing configuration yields 1.96 times the negative stiffness for an equivalent magnet volume compared to the design in Figure 1c (see Appendix D of the Supporting Information). This design addresses the dimensional constraints historically associated with broadband, low‐frequency noise control, establishing a foundation for compact solutions in space‐critical fields such as aerospace and consumer electronics.

Nanopores, such as those within activated carbon [25, 26], also exhibit similar negative stiffness, arising from the non‐monotonic van der Waals potential in confined spaces. However, this approach faces practical limitations, including challenges with particle size control, uniform pore size distribution, strong intrinsic nonlinearity, and long‐term acoustic instability [27]. In contrast, magnetic negative stiffness operates in a macroscopically ordered regime that delivers significantly stronger stiffness. It also offers in‐situ adjustability, mature nonlinear modeling, and robust environmental adaptability, making it an ideal experimental platform for engineering applications [28].

Negative stiffness has been extensively explored for vibration isolation, with key developments over the past two decades [29, 30, 31, 32, 33] following Molyneux's initial concept in 1957 [34]. Although researchers reported the first successful magnetic negative stiffness design for vibration isolation in 1994 [35], it initially garnered little attention even within the vibration control community. Robertson et al. [36] established a complete modeling framework for magnetic quasi‐zero‐stiffness isolators in 2009. However, these vibration isolation systems operate with millimeter‐scale displacements and require tuning to specific static loads [37, 38, 39]. In contrast, acoustic vibration amplitudes are minute: even under intense noise of 120 dB at 20 Hz, the oscillation amplitude of air particles remains below 0.54 mm, well below normal vibration isolation applications. Furthermore, acoustic absorbers have no static loading. These factors make the negative stiffness mechanism uniquely suited for low‐frequency sound absorption, where small pressure fluctuations drive substantial structural response with a very low activation energy barrier, forming an acoustic potential well (as illustrated in Figure 1e).

In what follows, Section 2 details the structure and operating principles of the aRMB and provides a theoretical analysis of its system performance. Section 3 presents experimental validation, Section 4 discusses the comparison of this research with previous studies, and Section 5 concludes the study.

## 2 Theoretical Analysis

This section details the structure and operating principles of the designed aRMB before presenting a comprehensive dynamic analysis. This analysis establishes the fundamental parameter relationships between vibration and acoustic systems. Furthermore, numerical calculations and simulation analyses investigate the stability of the bearing magnets across three degrees of freedom and evaluate the overall performance of the magnetic bearings.

### 2.1 Structural Design and Fundamental Dynamics Theory

Figure 2a illustrates the experimental setup for normal incidence sound. A loudspeaker placed at the left end of the impedance tube serves as the testing sound source, while the aRMB, equipped with built‐in permanent magnets, acts as a sound absorber at the opposite end. The interface between the incident sound and the absorber is similar to a loudspeaker diaphragm in that it has a rigid moving part connected to the impedance tube walls via a soft spider ring. This diaphragm is rigidly connected to the moving magnet and rigid rod assembly, which inserts into a mechanical ball bearing to facilitate axial motion under incident sound. The entire moving assembly acts a piston with a total mass *m_p_* = 20.6 g.

**FIGURE 2:**
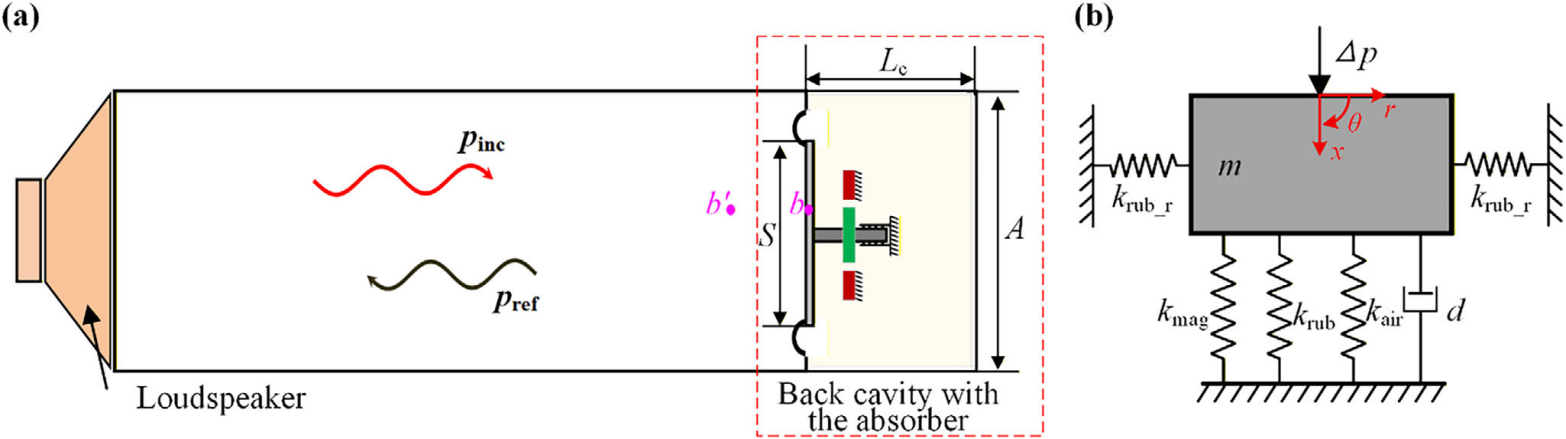
(a) Schematic of the normal incidence experimental setup featuring the aRMB. (b) Physical model of the aRMB's main body.

The interaction between the magnetic fields of the free and fixed magnets creates both axial and angular instability. While axial instability provides the required negative stiffness for this design, angular instability, which causes the free magnet to tilt, is undesirable. Inserting the steel rod into the linear ball bearing resolves this issue. Although this solution introduces friction, subsequent analysis under different sound pressure levels (SPLs) in Appendix E of the Supporting Information reveals that the vibration displacement amplitude is only a few microns to a few tens of microns; at such microscopic oscillations, the bearing friction is dominated by rolling within the grease film, which behaves as nearly linear viscous damping with a constant system damping. This contrasts with the classical Coulomb dry friction which is amplitude dependent. As a result, the bearing‐induced damping does not introduce any measurable amplitude dependence, and the absorption performance remains stable. Because the total damping stays essentially constant in both the with‐ and without‐negative‐stiffness configurations, this added friction does not affect our investigation into the influence of negative stiffness on the absorption bandwidth.

The abstract physical model (Figure 2b) depicts the moving body as an axisymmetric piston mass. Its governing dynamic equation is expressed as:

(1)

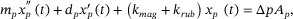

where primes denote time derivatives, the subscript $`p^{\prime }$ denotes the ‘piston’, and *x_p_* (*t*) is the positive displacement of the piston into the cavity. Furthermore, *m_p_* is the piston mass, *d_p_* is the piston damping, *k_mag_* < 0 is the negative magnetic stiffness acting on the piston, *k_rub_* is the ideally small parasitic stiffness caused by the rubber spider ring, *A_p_* is the effective oscillating area of the piston, and Δ*p* is the pressure difference between the two sides of the piston:

(2) $$\Delta p=p_{int}\;-p_{cav},\;p_{int}=p_{inc}\;-\rho_{0}c_{0}x_{p}^{\prime }\left(t\right)A_{p}/A_{d},$$

where the subscript $`int^{\prime }$ represents the interface position immediately in front of the piston (labelled as point $b^{\prime }$ in Figure 2a), $`cav^{\prime }$ represents the cavity, and $`inc^{\prime }$ denotes the ‘incident’ wave. As the piston moves into the cavity with velocity $x_{p}^{\prime }\left(t\right)$, it rarefies the interface air, generating a negative radiation pressure of $-\rho_{0}c_{0}x_{int}^{\prime }\left(t\right)$. Here, the interface velocity averaged over the duct cross section *A_d_*, $x_{int}^{\prime }$, relates to the piston velocity via the area ratio: $x_{p}^{\prime }\left(t\right)\;A_{p}=x_{int}^{\prime }\;\left(t\right)A_{d}$. The constants ρ_0_ = 1.225 kg/m^3^ and *c*_0_ =  343 m/s represent the density and speed of sound in air, respectively.

Applying mass conservation inside the cavity yields the air density (ρ) variation: $\rho_{0}x_{p}^{\prime }\left(t\right)\;A_{p}=V\partial \rho /\partial t$, where *V* is the cavity's air volume. Isentropic thermodynamics relates this density variation to the cavity pressure variation: $\partial \rho /\partial t=c_{0}^{-2}\;\partial p_{cav}/\partial t$. Assuming a harmonic time variation of *e* ^iω*t*^ (for which ∂/∂*t*  =  iω), we can formulate the final cavity air stiffness expression:

(3) $$p_{cav}=k_{cav}\;x_{p}\left(t\right)/A_{p},\;k_{cav}=\rho_{0}\;c_{0}^{2}A_{p}^{2}/V$$

Substituting Equations (2) and (3) into Equation (1) yields:

(4) $$m_{p}{x^{\prime \prime }}_{p}\left(t\right)+d_{p}{x^{\prime }}_{p}\left(t\right)+\underset{k_{\mathrm{tot}}}{\underset{︸}{\left(k_{cav}+k_{rub}+k_{mag}\right)}}x_{p}\;\left(t\right)=p_{int}\;A_{p}$$

Replacing the interface pressure *p_int_* on the right‐hand side with the incident pressure *p_inc_* adds radiation pressure to the system damping on the left‐hand side. This forms the full damping term, $\left(d_{p}+\rho_{0}c_{0}A_{p}^{2}/A_{d}\right)x_{p}^{\prime }\left(t\right)$, which is essential for later evaluations of whether acoustic excitation over‐damps the system. The radiation damping includes a geometrical factor of $A_{p}^{2}/A_{d}$, or (*A_p_*/*A_d_*)^2^ in pure dimensionless form, which appears frequently in subsequent terms. This factor derives from two physical effects: piston motion causes less volume displacement because *A_p_* < *A_d_*, and the resulting air pressure acts specifically on the piston surface *A_p_* to drive the response.

Reflection at the interface, averaged over the duct cross section, governs sound absorption. Rewriting Equation (4) in terms of the interface velocity $u_{int}\;\left(t\right)=x_{p}^{\prime }\;\left(t\right)A_{p}/A_{d}$, and transferring all time‐domain variables into frequency‐domain variables denoted by a hat: ${\hat{u}}_{int}\left(\omega \right)$ for time domain velocity ${\hat{u}}_{int}e^{i\omega t}$, frequency‐domain displacement $\hat{x}={\hat{u}}_{int}\;/i\omega$ and acceleration ${\hat{u}}_{int}i\omega$, yields $\left(m_{p}i\omega +d_{p}+k_{tot}/i\omega \right)\left(A_{d}/A_{p}\right)\;{\hat{u}}_{int}={\hat{p}}_{int}\;A_{p}$, where *k_tot_* = *k_cav_*  + *k_rub_* + *k_mag_* is the total stiffness felt by the piston. The complex pressure‐velocity ratio ${\hat{p}}_{int}/{\hat{u}}_{int}$ forms the acoustic impedance, which when normalized against ρ_0_ *c*_0_, becomes:

(5) $$Z_{int}\equiv \;\frac{{\hat{p}}_{int}}{\rho_{0}c_{0}{\hat{u}}_{int}}=\frac{m_{p}i\omega +d_{p}+k_{tot}/i\omega }{\rho_{0}c_{0}A_{p}^{2}/A_{d}}\;$$

Substituting *k_cav_* from Equation (3) into *k_tot_* in Equation (5) reveals its contribution to *Z_int_* as *c*_0_ *A_d_*/(iω*V*). This term is free from *A_p_* and only related to the effective air depth *V*/ *A_d_* = *L_v_* in the more obvious dimensionless form (i*k*_0_ *L_v_*)^−1^, where *k*_0_ =  ω/*c*_0_ is the wavenumber. Consequently, the air cavity stiffness depends entirely on the ratio of the wavelength to the volume‐based length *L_v_*, a metric that, unlike in simple rectangular or cylindrical cavities, does not correspond to a straightforward physical depth in complex resonator designs. Specifically, *L_v_* is the effective air height, defined as the net air volume divided by the tube area, while *L_c_* denotes the physical cavity depth of the actual cavity depth.

Since the air cavity stiffness is simply related to volume, the contributions of the magnetic gear (which includes the rubber spider‐ring), *k_mag_* + *k_rub_*, can be normalized by the stiffness of a pure air‐filled cavity using the ratio:

(6) $$\kappa =\frac{k_{mag}+k_{rub}}{k_{cav}}=\frac{\left(k_{mag}+k_{rub}\right)V}{\rho_{0}c_{0}^{2}A_{p}^{2}}\;$$

Rewriting the impedance in Equation (5) yields the following normalized form:

(7) $$\begin{array}{cc} & Z_{int}=ik_{0}L_{v}M+D+\frac{1+\kappa }{ik_{0}L_{v}},\;D=\frac{d_{p}A_{d}}{\rho_{0}c_{0}A_{p}^{2}}\;,\\ & M=\frac{m_{p}}{\rho_{0}V}\;{\left(\frac{A_{d}}{A_{p}}\right)}^{2},\;L_{v}=\frac{V}{A_{d}}\; \end{array}$$

Here, *D* represents the dimensionless piston damping arising from the rigid rod's friction against the ball‐bearing and the rubber spider‐ring's effect. Furthermore, *M* is the normalized piston mass, and both parameters are amplified by the previously explained factor (*A_d_*/*A_p_*)^2^. Since the effective oscillating area of the piston *A_p_* cannot be directly measured, 3D acoustic‐solid coupling simulations determine this amplification factor (see Appendix C of the Supporting Information). For the specific test rig, this yields *A_p_* =  π × (0.04046)^2^m^2^ and an amplification factor of (*A_d_*/*A_p_*)^2^ ≈ 4.14. It is more convenient to express the interface impedance in terms of a constant Γ and the dimensionless frequency *f*_1_:

(8) $$\begin{array}{cc} & Z_{int}=D+i\Gamma \left(f_{1}-f_{1}^{-1}\right),\;\;f_{1}=\frac{\omega }{\omega_{res}}\;,\;\;\omega_{res}=\frac{c_{0}}{L_{v}}\;\sqrt{\frac{1+\kappa }{M}},\\ & \Gamma =\sqrt{M\left(1+\kappa \right)}\;, \end{array}$$

where setting Im{Zint}=0 determines the resonance frequency ω_*res*_. The interface impedance *Z_int_* yields the sound absorption coefficient by:

(9) $$\begin{array}{cc} & \alpha =1-\;{\left|\frac{Z_{int}-1}{Z_{int}+1}\right|}^{2}=\frac{4D}{{\left(1+D\right)}^{2}+\Gamma^{2}f_{2}}\;,\\ & \alpha^{-1}=\frac{{\left(1+D\right)}^{2}}{4D}+\frac{\Gamma^{2}}{4D}f_{2},\;f_{2}\equiv {\left(f_{1}-f_{1}^{-1}\right)}^{2}. \end{array}$$

The absorption coefficient α typically exhibits an asymmetrical dome‐shaped curve near the resonance frequency in a linear frequency coordinate. To accurately obtain the peak position, the spectrum may be plotted on a logarithmic frequency scale where a parabolic curve can be fitted around the peak. This precisely identifies the dimensional peak position ω_*res*_ to obtain the dimensionless frequency *f*_1_. Then, Equation (9) implies a linear relationship for 1/α with respect to 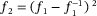. The range in which such linearity holds indicates the frequency range where the data quality is acceptable. Appendix A of the Supporting Information presents the linear fitting results for the experimental data. Data quality may suffer from two factors: measurement errors arising in the low frequency region where the microphone separation is inadequate for long waves, and high frequency deviations associated with non‐piston behavior.

Assuming the fitting yields $\alpha^{-1}=\alpha_{max}^{-1}+a_{1}f_{2}$, where the parabolic fit around the peak provides α_*max*_. The relationship α_*max*_ =  4*D*/(1 + *D*)^2^ allows two solutions for dimensionless damping:

(10) $$D=\left(2/\alpha_{max}-1\right)\pm \sqrt{{\left(2/\alpha_{max}-1\right)}^{2}-1}.$$

Experimental data providing the phase relation at the resonance frequency determines the correct solution. More specifically, the complex reflection coefficient at the interface position is ${\hat{p}}_{ref}/\;{\hat{p}}_{inc}=\left(D-1\right)/\left(D+1\right)$. An in‐phase relation validates the larger damping solution; otherwise, the lower damping solution applies. Once *D* is determined, utilizing the slope of the curve fit Γ^2^/(4*D*) (cf. Equation (9)) yields Γ. Simultaneously solving the system of equations formed by the definitions of ω_*res*_ and Γ in Equation (8) then determines *M* and κ:

(11) $$M=\Gamma /\left(k_{0}L_{v}\right),\;\kappa =\left(k_{0}L_{v}\right)\Gamma -1.$$

Notably, published papers providing only the α curve without further experimental information cannot uniquely resolve the choice of damping and the resulting crucial stiffness parameter κ. For our own experiment, we found that the larger damping solution prevails.

While this article focuses on the impact of the negative stiffness factor, −1 < κ < 0, on sound absorption performance, the level of damping is also crucial. Beyond the normalized damping parameter *D* defined in Equation (7), damping should also be viewed from the perspective of the system response to the incident wave pressure *p_inc_*. Replacing the interface pressure *p_int_* with $p_{inc}-\rho_{0}c_{0}\left(A_{p}^{2}/A_{d}\right)x_{p}^{\prime }\left(t\right)$ in Equation (4) transforms the system into a single‐degree‐of‐freedom oscillator with mass *m_p_*, total damping $\;d_{tot}=d_{p}+\rho_{0}c_{0}\left(A_{p}^{2}/A_{d}\right)$ and stiffness *k_tot_*. This yields a natural frequency of $\omega_{n}=\sqrt{k_{tot}/m_{p}}$ and a dimensionless damping ratio of $\zeta =d_{tot}\;/\left(2\sqrt{m_{p}k_{tot}}\right)$. The actual oscillation frequency is $\omega_{n}\sqrt{1-\zeta^{2}}$. In terms of the parameters defined in Equations (6) and (7), the damping ratio is written as:

(12) $$\zeta =\frac{D+1}{2\sqrt{M\left(1+\kappa \right)}}\;.$$

When ζ > 1, the system is overdamped and will not produce oscillation for a transient disturbance (while the response to forced sound incidence will peak at frequency ω_*res*_ unrelated to damping). When κ approaches −1, the denominator approaches zero ensuring ζ > 1.

### 2.2 Magnetic Characteristic Analysis

In traditional face‐to‐face magnets, negative stiffness is generated via magnetic attraction, meaning the air gap between magnets inherently changes with their displacement. In contrast, the negative stiffness of bearing magnets arises from the repulsive force between magnetic rings. Consequently, this air gap (i.e., the radial clearance between the rings) remains fixed during axial displacement [40]. Theoretically, because a smaller air gap yields a stronger repulsive force, the negative stiffness from bearing magnets can increase infinitely. Furthermore, the initial operating point of the bearing magnets corresponds to both the maximum negative stiffness and the equilibrium position. These characteristics make bearing magnets the optimal choice for in‐depth study and experimental design.

Based on the detailed derivation in Appendix D of the Supporting Information, Figure 3 plots the bearing magnets' force/torque and stiffness curves across three degrees of freedom: axial (*x*‐direction), radial (*r*‐direction), and angular (θ‐direction). Table 1 details the specific dimensions for the three types of bearing magnets used in the experiments.

**FIGURE 3:**
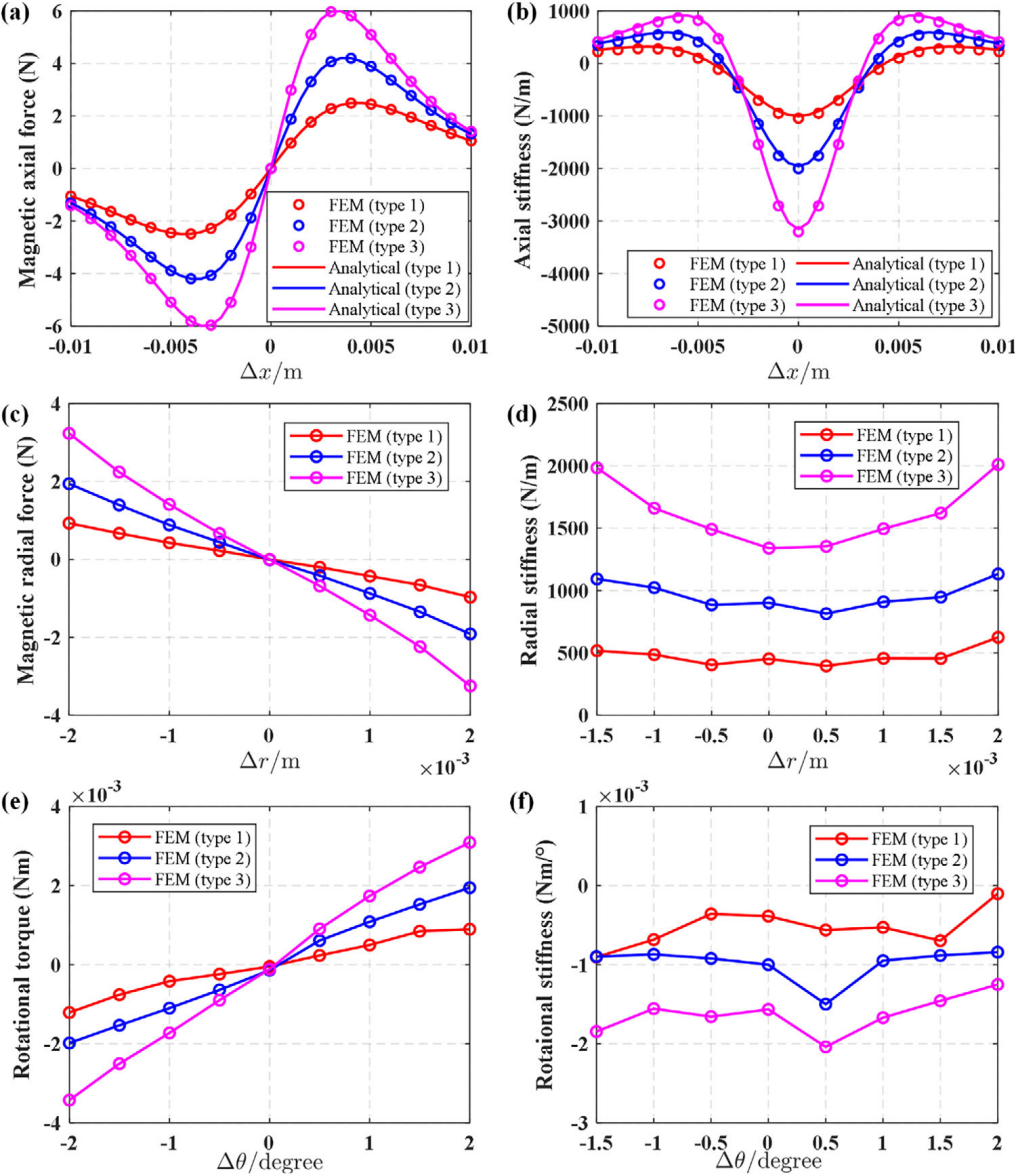
Stability calculation for the three types of bearing magnets using the finite element method (FEM) and analytical methods. (a) Magnetic axial force and (b) axial stiffness. (c) Magnetic radial force and (d) radial stiffness. (e) Magnetic rotational torque and (f) rotational stiffness.

**TABLE 1:** Specific dimensions of the three bearing magnet types.

Ring magnets	Types	Inside radius (mm) × external radius (mm) × thickness (mm)
Inner magnet (moving)	Types 1–3	2 × 9 × 3
Outer magnet (fixed)	Type 1	15 × 20 × 3
	Type 2	13.5 × 20 × 3
	Type 3	12.5 × 20 × 3

Figure 3a,b illustrates the axial force and stiffness of the bearing magnet under different axial displacement *x*. The axial magnetic force is zero at the equilibrium position *x*  =  0, where the axial negative stiffness peaks. For positive and negative displacements (*x* > 0 and *x* <  0 respectively), the inner free magnet experiences an increasing repulsive force in the direction of displacement, accompanied by rapidly decreasing negative stiffness. Beyond a certain displacement from equilibrium (approximately *x*  =   ±3.5 mm), the stiffness becomes positive, thereby limiting the effective operating range of the acoustic resonator. Magnets with smaller air gaps exhibit larger negative stiffness (see type 3 in Figure 3b); consequently, decreasing the air gap may greatly improve magnetic negative stiffness in practice. In summary, optimizing acoustic absorption performance requires operating the resonator as close as possible to the equilibrium position while maintaining a reasonably small air gap between the magnets. Figure 3c,e presents the radial force and stiffness characteristics of the inner bearing magnet. The radial force exhibits a nonlinear decrease with increasing radial displacement, Δ*r*. Specifically, increasing Δ*r* positively generates a corresponding restoring force in the opposite (‐*r*) direction. This behavior, directly evidenced by the consistently positive radial stiffness, confirms the structure's radial stability. Conversely, the inner ring magnet's angular torque and stiffness (Figure 3e,f) reveal significant instability in the tilt direction. This angular instability leads to increased tension, thereby introducing unintended (parasitic) axial stiffness.

To solve this problem, the rolling contact between the moving steel rod and a linear ball bearing constrains the angular degree of freedom, preserves the essential axial negative stiffness while ensuring the moving body vibrates freely along the intended single axis. Based on the generated rotational torque (Figure 3e), the magnitude of magnetic torque in the range −2° < θ < 2° is less than 4 × 10^−3^ Nm. The equation *F_r_* = *M_mag_* /*L_rod_* determines the radial force exerted by this magnetic torque on the linear bearing, where *F_r_* is the radial force, *M_mag_* is the magnetic torque, and *L_rod_* is the steel rod length. Given the rod length of *L_rod_* =  2.6 cm, the calculated radial force is *F_r_* =  0.154 N. Because the linear bearing (MYT Brand, Model: LMF5UU) has a maximum radial dynamic loading of *F*_*r*, *max*_ =  261N, it operates well within its capacity, validating this design solution. To further verify this design, Appendix B of the Supporting Information presents an eigenmode analysis of the entire moving assembly (oscillating plate, rubber‐spider ring, steel rod, and inner magnet).

## 3 Experimental Validation

Figure 4a displays the experimental setup for measuring sound absorption with the aRMB, while Figure 4b details its 3D structure. A computer generates the digital source signal, which a digital‐to‐analogue converter (DAC) module converts into analog electric signals; a power amplifier then amplifies these signals to drive the source loudspeaker. Microphones 1 and 2 collect the resulting sound signals, which are amplified and digitized by an analogue‐to‐digital converter (ADC) module, and fed back to the computer for data analysis. The analysis follows the established two‐microphone transfer function method proposed by Chung and Blaser (1980) [41], which decomposes the acoustic field into its incident and reflected components using a transfer function relation between the two microphone signals. This decomposition allows for the determination of the test sample's complex reflection coefficient, acoustic impedance, and sound absorption coefficient. The microphone positions for the two‐microphone transfer function method are as follows: the two microphones are spaced 0.08 m apart, and the distance from the sample surface to the microphone that is closer to the sound source is 0.31 m. The impedance tube has a square cross‐section with inner side length of 0.102 m, yielding an area *A*_d_ =  (0.102 m)^2^.

**FIGURE 4:**
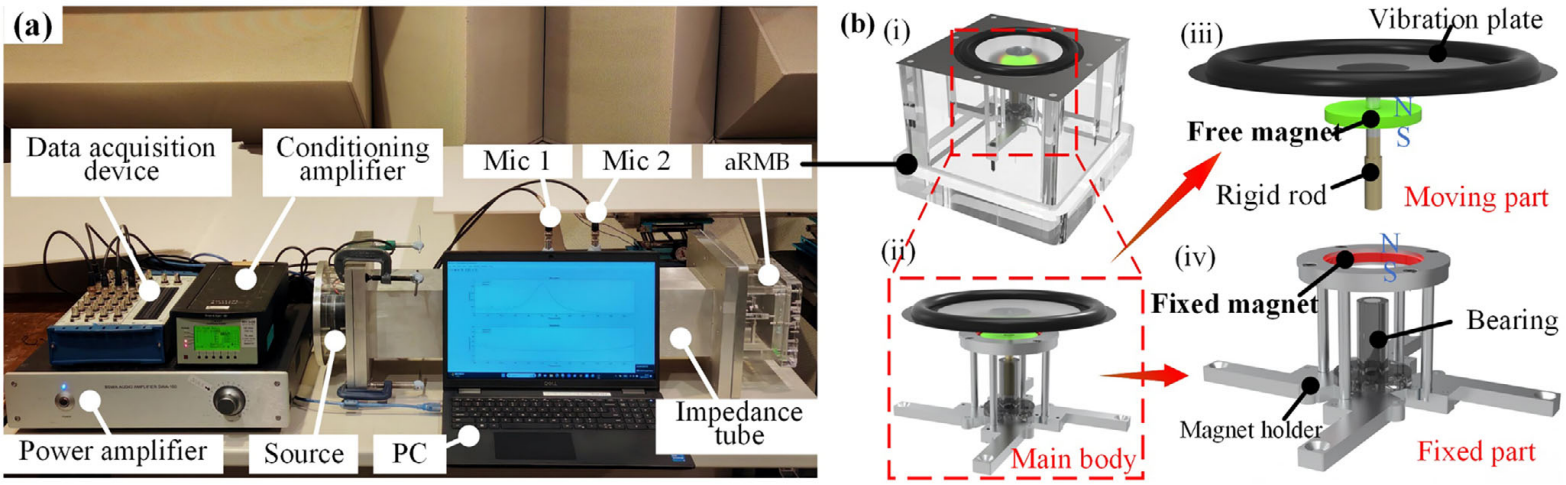
(a) Experimental setup for measuring sound absorption under normal incidence conditions. (b) Detailed 3D structure of the aRMB used in the tests.

The system's overall positive stiffness comprises contributions from the cavity air and the rubber ring. To first calculate the rubber ring's equivalent stiffness *k_rub_*, we measured the resonant frequency from the normal incidence sound absorption with an open cavity (Figure 5a) to characterize the pure rubber stiffness condition. The test results (Figure 5b) indicate a resonant frequency of *f*_res_ = 33.6 Hz. Given a measured total mass of *m_p_* =  20.6 g, the equation $f_{\mathrm{res}}=\frac{1}{2\pi }\;\sqrt{k_{rub}/m_{p}}$ yields a calculated stiffness of *k_rub_* =  918 N/m.

**FIGURE 5:**
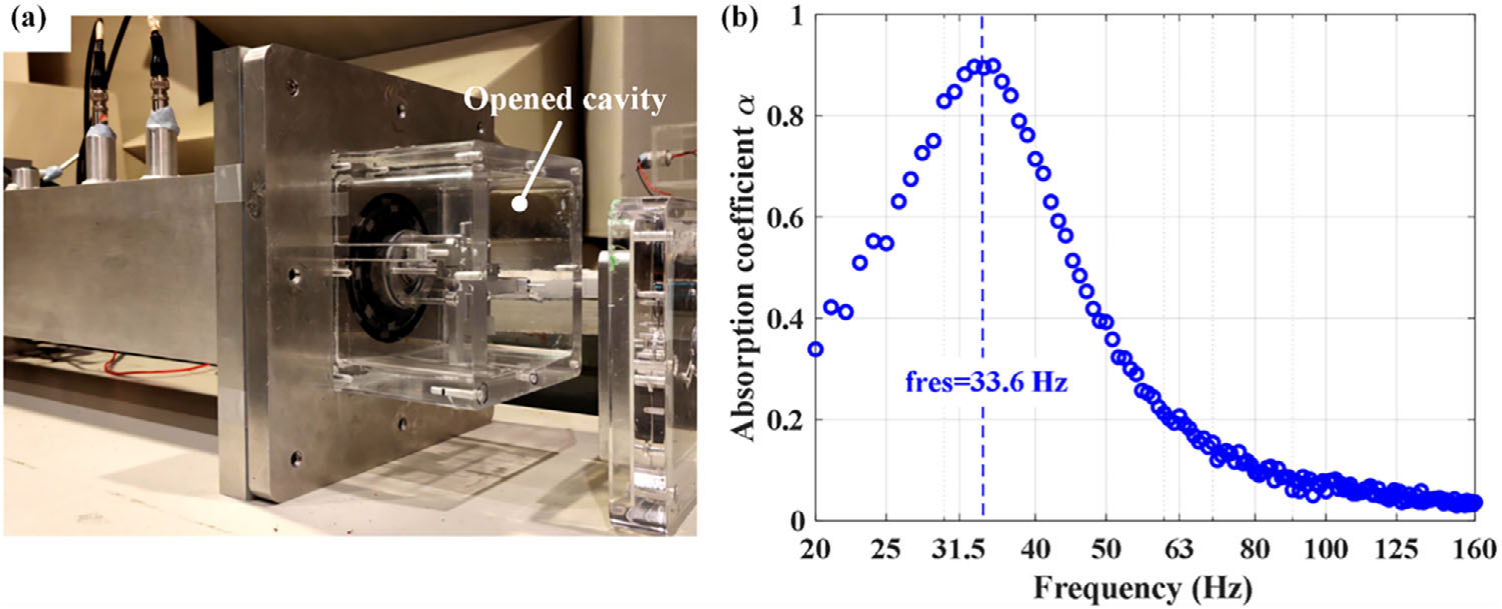
(a) Open cavity condition used to obtain the rubber suspension system's stiffness *k*_rub_. (b) Measured absorption coefficient curve under the open cavity condition.

Determining the air stiffness, $k_{cav}=\rho_{0}\;c_{0}^{2}A_{p}^{2}/V$, requires the effective volume of the cavity. Because the internal mechanical structure (e.g., bearing magnets) occupies physical space, its volume must be subtracted from the overall cavity volume. Using CAD software, we calculate the mechanism's volume at approximately 32.8 cm^3^. For a cavity with a physical depth of *L_c_* =  90 mm, ascertaining the net cavity volume yields an air stiffness of *k_cav_* =  4219 N/m. Consequently, the theoretical total positive stiffness *k*_+_ = *k_rub_*  +  *k_cav_* =  5137 N/m. Furthermore, Equation (6) yields a theoretical initial stiffness ratio of κ_0_ =  0.22 for the pure resonator *k_mag_* =  0.

Figure 6 displays the measured impedance for the 90 mm cavity. In the low‐frequency band, the resistance remains fairly constant at around *D*  =  2.2 under different negative magnetic stiffness. To understand the source of damping mechanisms, we conducted a separate experiment without the mechanical ball‐bearing, which yielded a dimensionless overall damping of 1.6. This implies that both ball bearing and the rubber suspension contribute to damping. Although the axial friction of the slider‐ball structure introduces a measurable increase in the total system damping (37%), the comparative experiments between the configurations with and without negative stiffness were performed under identical mechanical constraints, and the measured damping levels remain essentially the same in both cases. In fact, a dimensionless damping higher than the ideal value of 1 broadens the bandwidth at the expense of the peak absorption, and it is sometimes desirable in practical problem depending on the exact goal of optimization. Having said these, it is perhaps more desirable to bring down the level of bearing damping by using air bearing in the future and leave the desired damping increase to means easier for adjustment. It has to be noted though that the current increase of 37% in damping does not affect the conclusion regarding the observed significant bandwidth broadening mainly attributed to the negative stiffness. Zero reactance indicates resonant frequencies of 79.1, 71.3, 62.6, and 57.8 Hz for the no‐magnet case and magnet types 1, 2, and 3, respectively. Because magnet types 1, 2, and 3 provide increasing negative stiffness, these results demonstrate that resonant frequency decreases as negative stiffness increases in magnitude.

**FIGURE 6:**
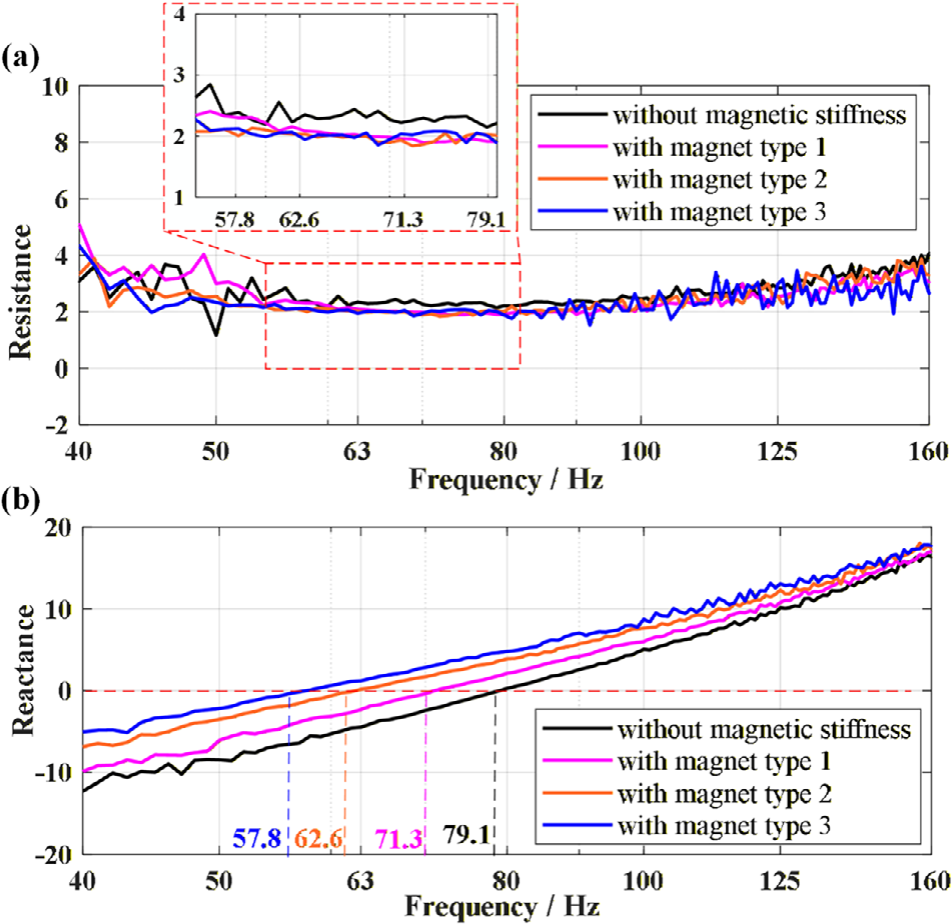
Measured normalized resistance (a) and reactance (b) of the aRMB with different magnet types using a 90 mm cavity. The configuration “without magnetic stiffness” implies the removal of fixed magnets while retaining the moving magnet so that all cases have the same moving mass.

Within the frequency range of 40–160 Hz, fitting a linearized reactance model to the measured data extracts the acoustic mass *M* and negative stiffness ratio κ. This process constructs an overdetermined linear system based on the normalized reactance relation *X*(*f*_1_)  =  *Mf*_1_ − (1 + κ)/*f*_1_, where *X*(*f*_1_) represents the imaginary part of the measured specific impedance. Solving this system via a least‐squares method (pseudo‐inversion) minimizes the fitting error and provides a numerically stable solution for the parameters *M* and κ. Here, the frequency‐dependent terms *f*_1_ and −1/*f*_1_ form the coefficient matrix, and the reactance values *X*(ω) constitute the observation vector. The stiffness ratio of the system measures 0.22 without magnetic negative stiffness, which is consistent with the theoretical initial positive stiffness κ_0_ obtained above. Under the action of the three magnet types, the stiffness ratio κ reduces to −0.01,   −0.25,   −0.35, respectively. The relative mass *M* for each case was found to be 76.9, 76.9, 75.7, 76.9, respectively. These measured values show good agreement with the theoretical value derived from *M*  = *m_p_* (*A_d_*/*A_p_*)^2^/ρ_0_ *V* using the weighed mass *m_p_* =  20.6 g. This demonstrates that introducing the fixed magnets (and thus the negative stiffness *k_mag_*) does not alter the mass in all four experiments shown in Figure 6. Therefore, the decrease in resonance frequency is caused purely by the decrease in overall stiffness, as opposed to an increase in overall acoustic mass.

Corresponding to Figures 6 and 7 illustrates the aRMB's sound absorption curves for a cavity depth of 90 mm under varying negative stiffness. Open circles represent the experimental data points, while solid lines denote their corresponding curve fits. As negative magnetic stiffness increases, the resonance frequency of the absorption coefficient curve decreases. Specifically, utilizing magnet type 3 reduces the stiffness ratio κ from 0.22 to −0.35 (where negative stiffness *k_mag_* ≈ −2456 N/m), shifting the resonance frequency from 79.1 to 57.8 Hz. Table 2 lists the relevant parameter values for the four experimental groups.

**FIGURE 7:**
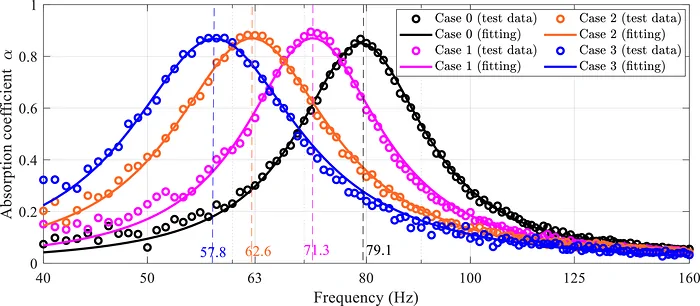
Sound absorption coefficient curves of the aRMB with different negative magnetic stiffness values for a 90 mm depth cavity. Case 0 (test data): without magnetic negative stiffness. Case 0 (fitting): *D*  =  2.06, *M*  =  76.9, κ  =  0.22, ζ  =  0.17. Case 1 (test data): with magnet type 1. Case 1 (fitting): *D*  =  2.12, *M*  =  76.9, κ  =   − 0.01, ζ  =  0.18. Case 2 (test data): with magnet type 2. Case 2 (fitting): *D*  =  2.12, *M*  =  75.7, κ  =   − 0.25, ζ  =  0.21. Case 3 (test data): with magnet type 3. Case 3 (fitting): *D*  =  2.14, *M*  =  76.9, κ  =   − 0.35, ζ  =  0.22.

**TABLE 2:** Relevant parameters for the four experimental groups with *L_c_* =  90 mm.

	*k_mag_* (N/m)	*k_tot_* (N/m)	*f*_res_ (Hz)	ζ	κ
No magnet	0	5142	79.1	0.17	0.22
Type 1	−969	4173	71.3	0.18	−0.01
Type 2	−1973	3169	62.6	0.21	−0.25
Type 3	−2456	2686	57.8	0.22	−0.35

Two separate criteria should assess the gain achieved by magnetic negative stiffness. First, introducing the fixed magnet reduces the total system stiffness *k_tot_* from 5142 to 2686 N/m, yielding a ratio of 1.91. However, a more stringent criterion is the reduction from *k_cav_* =  4219 N/m, which represents the state without the diaphragm suspension. This comparison yields a ratio of 4219/2686 = 1.57. Previous literature utilizing negative magnetic stiffness lacks this stringent and meaningful assessment. This omission occurs because the suspension is only needed when the magnetic design enforces piston‐mode vibration, but it is not needed in classical designs. In these classical designs, the system stiffness is *k_cav_* without any parasitic stiffness, and engineers simply add acoustic mass via a perforated panel to bring down the system resonance frequency to the desired value. Based on the second criterion, the current study is the first demonstration that magnetic negative stiffness has achieved a breakthrough when compared with classic designs [42]. This classic approach is briefly summarized below for completeness.

Among classic resonator designs, a cavity covered by a perforated panel is the most straightforward. Forcing air through small apertures generates damping. To quantify this, Maa's [43] widely used aperture impedance approximation is listed below alongside the interface impedance *Z_int_* needed to obtain the sound absorption coefficient α:

(13) $$Z_{int}=\frac{\left(Z_{r}+iZ_{i}\right)}{\sigma }+\frac{1}{i\;\tan\left(\omega L_{v}/c_{0}\right)},\;\alpha =1-{\left|\frac{Z_{int}-1}{Z_{int}+1}\right|}^{2}$$

(14) $$Z_{r}=\frac{32\nu t_{p}}{a^{2}c_{0}}\;\sqrt{1+\frac{K_{abl}^{2}}{32}}+\frac{4}{c_{0}}\sqrt{\frac{\omega \nu }{2}},\;K_{abl}^{2}=\frac{a^{2}\omega }{4\nu }\;,$$

(15) $$Z_{i}=t_{p}\;\frac{\omega }{c_{0}}\left(1+\frac{1}{\sqrt{9+K_{abl}^{2}/2}}\right)+0.85a\frac{\omega }{c_{0}}.$$

where *t_p_* is the panel thickness, *a* is the perforation diameter, σ is the perforation ratio, ν is the kinematic viscosity of air, ω is the angular frequency, *L_v_* is the effective cavity depth, and *K_abl_* is the ratio of the aperture diameter to the acoustic boundary layer thickness. Because researchers have extensively studied the perforated panel cavity and established its experimentally validated theoretical model, this work directly employs these classic analytical formulas for further optimization studies rather than conducting new experiments.

Since aperture resistance is inversely proportional to the square of the aperture diameter, perforated panels can implement a very wide range of damping. Meanwhile, fast air motion through the holes and sideways motion outside the apertures and over the impervious panel walls add to the acoustic mass, which approximately equals the mass of an air column of height (*t_p_* + 0.85*a*)/σ. By choosing an appropriate diameter and porosity, engineers can obtain most desired combinations of damping *D* and acoustic mass *M*.

Han et al. [42] explained the optimization of these classic designs, demonstrating that optimal dual‐cavity classic designs outperform most acoustic meta‐materials (AMMs) found to date. There are two main reasons why existing AMMs fail to deliver better performance than simple classic designs. First, for each specific incident noise spectrum, there exists an optimal acoustic mass needed to align system resonance with the energy‐weighted center frequency of the incident noise. Many in the past have pursued the so‐called “deep sub‐wavelength” instead of the most critical wavelength. A lot of AMM designs add acoustic mass through complex construction, often leading to a mass higher than the optimal value. Second, most existing AMMs do not change system stiffness, and some may even increase it, similar to our earlier attempts with magnetic negative stiffness designs. When the system stiffness is reduced, the optimal added mass will also be lower, both contributing to better broadband performance. Since the device in this study delivered the first working negative stiffness via magnetic fields, it has the potential to produce a new class of AMM.

To demonstrate the stability and applicability of the designed aRMB, we conducted experiments with various cavity depths. As an example, Figure 8 compares the sound absorption curves for four cases with a 70 mm cavity depth to illustrate this experimental breakthrough. (Appendix F of the Supporting Information provides details for other cavity depths.) Open circles represent experimental data, and solid lines show the fitted absorption coefficient α based on a simple resonator model with constant mass, damping, and stiffness. The black curves correspond to the configuration without magnetic stiffness, while the blue curves represent results with magnet type 3. This comparison illustrates the shift in resonance frequency caused by reducing the system stiffness alone while the mass remains constant. Specifically, the resonance frequency decreases from 85.6 Hz (without magnetic stiffness) to 62.8 Hz (with magnet type 3), κ drops from 0.13 to −0.40, and *M* changes only slightly from 103.1 to 100.8. This indicates that the introduction of magnetic negative stiffness has exceeded the thermodynamics limit, reaching κ  =   −0.4. This method of achieving low‐frequency sound absorption by introducing negative stiffness is referred to as stiffness tuning. To compare stiffness tuning with the classic mass‐tuning approach, we consider the two fundamental strategies under the constraint of identical back‐cavity volume (i.e., identical air stiffness) and targeting the same low‐frequency resonance. The perforated panel serves only as a concrete experimental realization of the mass‐tuning strategy, providing the required acoustic mass and damping.

**FIGURE 8:**
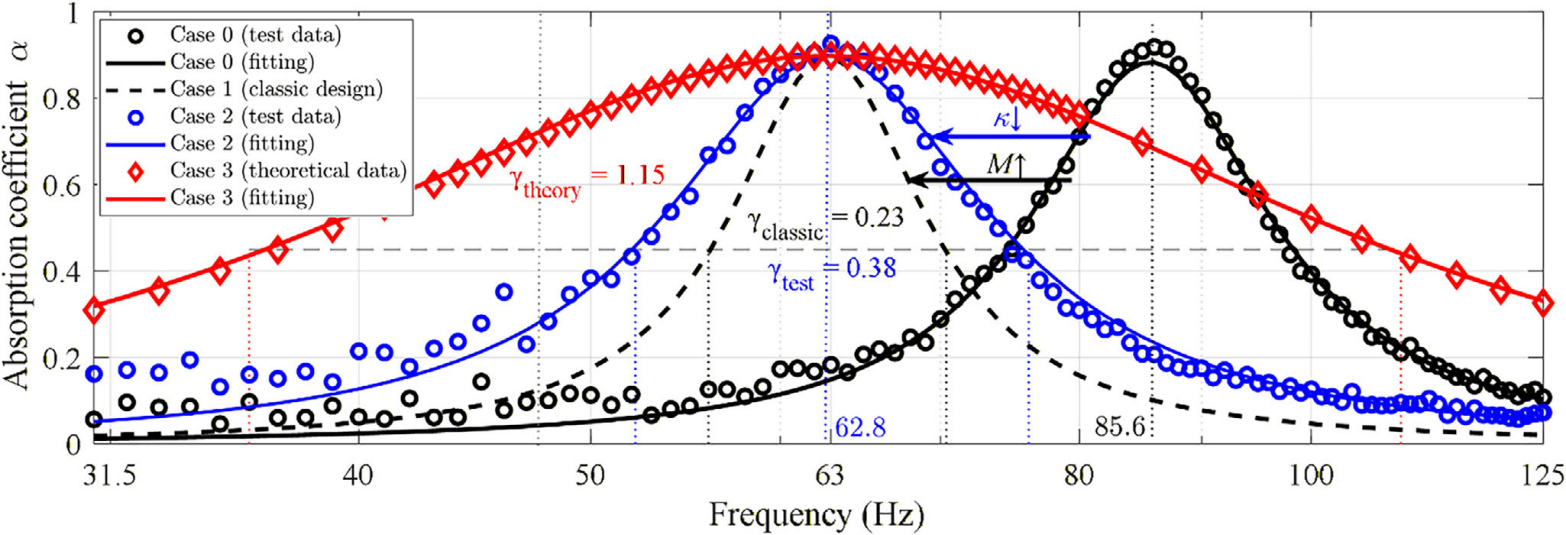
Comparison of absorption coefficient curves between the aRMB design and the classic perforated‐panel design covering a pure air cavity at *L*_c_ =  70 mm in the two lowest octaves (31–125 Hz). Case 0 (test data): without magnetic stiffness. Case 0 (fitting): *I*  =  0.32, *D*  =  2.04, *M*  =  103.1, κ  =  0.13, where I is the absorption integral defined in Equation (16). Case 1 (classic design): *I*  =  0.28, *D*  =  1.94, *M*  =  169.1,κ  =  0. Case 2 (test data): *L_c_* =  70 mm with magnet type 3. Case 2 (fitting): *I*  =  0.44, *D*  =  1.94, *M*  =  100.8, κ  =   −0.40. Case 3 (theoretical data): simulation result of the advanced design. Case 3 (fitting): *I*  =  0.90, *D*  =  1.94, *M*  =  33.6, κ  =   −0.80.

For the magnetic‐stiffness configuration at *L_c_* =  70 mm, we now consider a classic perforated‐panel design relying only on the cavity stiffness (*k_cav_* =  5481 N/m), tuned to achieve the same resonance frequency (62.8 Hz) by increased mass and the same dimensionless overall damping (*D*  =  1.94) as the magnet design. The panel parameters (black dashed line in Figure 8) are: aperture diameter *a*  =  3.68 mm,  porosity σ  =  0.21% and panel thickness *t_p_* =  17 mm, yielding damping *D*  =  1.94, mass *M*  =  169.1, stiffness ratio κ  =  0 (indicating no structural stiffness, i.e., *k_rub_* =  0). This configuration yields an absorption curve that represents the classic mass‐tuning strategy under the thermodynamics limit.

Two metrics evaluate sound absorption performance: the effective sound absorption bandwidth and the total sound absorption. The quality factor, defined as γ  =  Δ*f*/*f*_res_ (where Δ*f* is the full width at half maximum absorption coefficient, and *f*_res_ is the resonant frequency) assesses the effective absorption bandwidth. A weighted integral characterizes the integrated sound absorption (*I*) in a frequency range:

(16) $$I=\int_{\omega_{1}}^{\omega_{2}}\alpha \left(\omega \right)/\omega d\omega =\int_{\ln\omega_{1}}^{\ln\omega_{2}}\alpha \left(\omega \right)d\left(\ln\omega \right)\;.$$

This formulation gives greater weight to low frequencies, making it especially suitable for assessing low‐frequency performance. The target frequency range spans the two lowest octaves (31–125 Hz). For the aRMB with *L_c_* =  70 mm and magnet type 3 (blue line in Figure 8), the experimental results show *I*  =  0.44 and γ  =  0.38. For the classic design at the same cavity depth, *I*  =  0.28 and γ  =  0.23. Therefore, the aRMB achieves 1.57 times the total absorption and 1.65 times the bandwidth of the classic design. The comparative experiments presented above were all conducted at a sound pressure level of 95 dB.

Since magnetic force is essentially nonlinear, it is necessary to establish the range in which aRMB can operate in a satisfactory linear manner and to determine the nonlinear behavior outside the linear range. To do so experimentally, the magnitude of the incident SPL was adjusted by the power amplifier. The results show that the measured absorption curves from 95 dB to 105 dB are nearly identical, with constant stiffness, mass, and damping. The test rig does not have capacity to operate beyond 105 dB, so the nonlinear behavior beyond this point is studied using numerical simulations. Details of the simulation, as well as the repetition of experiments up to 105 dB, are given in Appendix E of the Supporting Information. It is found that nonlinear behavior begins to become noticeable in the absorption curve from around 125 dB. When the incident sound exceeds 127 dB, the displacement of the magnetic piece goes beyond a critical position of no return. In other words, the current prototype with the Type 3 magnetic bearing functions satisfactorily up to about 125 dB, a level which causes human hearing pain.

Taken together, the broadband absorption improvement over the classic design and the stable operation up to 127 dB constitute a convincing proof of concept for low‐frequency sound absorption enabled by effective magnetic negative stiffness. Compared to the classic design, the aRMB improves the total sound absorption capacity and broadens the bandwidth. However, because technical issues rather than fundamental principles limit the current experiments, the potential of magnetic negative stiffness extends considerably further. Optimizing structural parameters can substantially enhance performance. For example, reducing the air gap between the bearing magnets from 3.5 mm (magnet type 3) to 2.5 mm increases the theoretical magnetic negative stiffness to −5541 N/m, thereby reducing 1 + κ from 0.6 to 0.2. Replacing the acrylic plate with a lightweight conical diaphragm (inspired by loudspeaker design, see Appendix B of the Supporting Information) ensures piston motion and leads to a threefold reduction of mass to *M*  =  33.6. In order to better evaluate the rationality of the parameters of this advanced design, a three‐dimensional simulation model was established. The red rhombus marks in Figure 8 represent the data points of the simulated sound absorption curve. Under these conditions, κ reaches −0.8, quality factor γ is further increased to 1.15, and the absorption integral *I* increases to 0.90. Relative to the classic design, this represents a 3.21‐fold increase in sound absorption capacity and a 5‐fold widening of bandwidth. A κ value of −0.80 is equivalent to increasing the effective cavity depth by 5 times. Meanwhile, the nonlinear effects of this model were also explored through simulation results, and the results show that this model exhibited stable sound absorption performance even at high incident sound pressure levels (SPL) up to 122 dB. The specific analysis content can be found in Appendix G of the Supporting Information. Although simulation verified its feasibility, this structural system still involves practical challenges. A smaller air gap narrows the effective working range, making the system highly sensitive to assembly tolerances and misalignment. Manufacturing an ideal lightweight conical diaphragm and integrating it with the moving assembly demands precision will not be easy; minor asymmetries can introduce higher‐order modes that degrade performance. These difficulties are of a technical in nature rather than scientific, and improving our technical design and manufacturing accuracy will be the focus of future work. The present study establishes the fundamental principle that a net magnetic negative stiffness can be achieved with a feasible set of design parameters. Overall, the experimental results already indicate that physical volume no longer limits the resonator's performance, paving the way for compact, high‐performance acoustic resonators.

## 4 Discussion

Figure 9a,b presents the fitting results with the test data in Ref. [22] (from Figure 5 therein) and Ref. [23] (from Figure 8a,c therein), respectively. By fitting the tested sound absorption curves shown in the paper, κ and *M* can be obtained by Equation (11) in Section 2.

**FIGURE 9:**
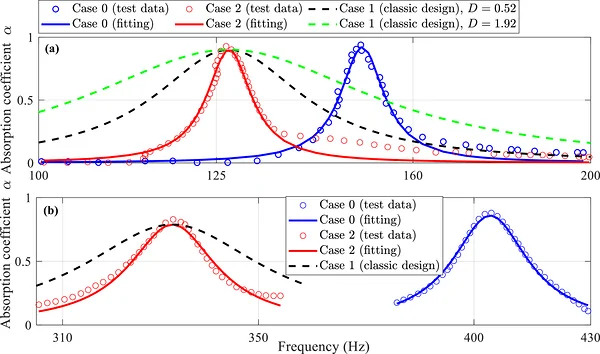
(a) The test data were obtained from Figure 5 from Ref. [22]. For *D* < 1. Case 0 (test data): without magnetic negative stiffness; Case 0 (fitting): *f_res_* =  150.1 Hz, *D*  =  0.56, *M*  =  132.7, κ  =  3.11. Case 1 (classic design): *f_res_* =  127 Hz, *D*  =  0.52, *M*  =  45.1, κ  =  0; Case 2 (test data): with negative stiffness; Case 2 (fitting): *f_res_* =  127 Hz, *D*  =  0.52, *M*  =  149.3, κ  =  2.31. For *D* > 1. Case 0 (test data): without magnetic negative stiffness; Case 0 (fitting): *f_res_* =  150.1 Hz, *D*  =  1.78, *M*  =  237.0, κ  =  6.34. Case 1 (classic design): *f_res_* =  127 Hz, *D*  =  1.92, *M*  =  45.1, κ  =  0; Case 2 (test data): with negative stiffness; Case 2 (fitting): *f_res_* =  127 Hz, *D*  =  1.92, *M*  =  286.9, κ  =  5.36. (b) The test data were obtained from Figure 8a,c from Ref. [23]. Case 0 (test data): without negative stiffness. Case 0 (fitting): *f_res_* =  404.0 Hz, *D*  =  0.45, *M*  =  577, κ  =  0.14; Case 1 (classic design): *f_res_* =  331.8 Hz, *D*  =  2.70, *M*  =  754.6, κ  =  0; Case 2 (test data): with negative stiffness; Case 2 (fitting): *f_res_* =  331.8 Hz, *D*  =  2.70, *M*  =  1549, κ  =  1.06.

Since no specific damping conditions were provided in the experiment in Ref. [22], two possibilities were analyzed separately: the dimensionless overall damping *D* > 1 and *D* < 1. When *D* < 1, magnetic negative stiffness brings κ down from 3.11 to 2.31. When *D* > 1, magnetic negative stiffness brings κ down from 6.34 to 5.36. In both damping conditions, κ remains positive despite being reduced. This means that the negative stiffness has played a role, but it merely offsets the additional positive stiffness values introduced by the system, and the final stiffness is still greater than the pure air stiffness of the cavity (part of the thermodynamics limit κ  =  0). That is to say, a net negative stiffness is yet to be achieved.

It is worth noting that Zhang et al. [23] presented a low‐frequency sound absorber in 2025 that achieves efficient absorption in the 332–404 Hz range by leveraging the negative stiffness of a buckling plate coupled with an air cavity. The fitted data are derived from Figure 8a (without negative stiffness) and Figure 8d (with negative stiffness) of that paper. Moreover, it is indicated in the paper that the damping in the tests for these two states is under‐damped and over‐damped, respectively. Therefore, through curve fitting, the values of κ and *M* can be accurately obtained; the fitting result is shown in Figure 9b of the present paper. The calculation shows that κ  =  0.14 without the negative stiffness system, whereas κ  =  1.06 when it is used, indicating that there was actually an increase in the system stiffness, apparently due to parasitic stiffness. Moreover, because the buckling plate has a relatively large modal mass under pre‐stress, the increase in mass led to a shift in the resonance frequency towards lower frequencies instead of being achieved by a net negative stiffness. Detailed comparison between our work and the findings with these two‐representative works [22, 23] is listed in Table 3.

**TABLE 3:** Comparison with representative prior studies on negative stiffness in compact resonators for acoustic absorption.

	Negative stiffness method	Without negative stiffness				With negative stiffness			
		*f*_res_ (Hz)	*D*	*M*	κ	*f*_res_ (Hz)	*D*	*M*	κ
Li et al. [22]	Symmetrical magnets	150.1	0.56	132.7	3.11	127	0.52	149.3	2.31
		150.1	1.78	237.0	6.34	127	1.92	286.9	5.36
Zhang et al. [23]	buckling plates	404.0	0.45	577	0.14	331.8	2.70	1549	1.06
This paper	Bearing magnets	85.6	2.04	103.1	**0.13**	62.8	1.94	100.8	**−0.40**

In summary, due to the aforementioned instability issues, effective magnetic negative stiffness has not yet been achieved in acoustics to date. The present work is the first to realize effective magnetic negative stiffness, attaining a stiffness ratio of κ  =   −0.4, beyond the thermodynamic limit of κ  =  0 for a pure air cavity and even the lower limit of − 2/7  =   −0.286 for a cavity filled with porous materials.

## 5 Conclusions

In this work, we introduced and experimentally validated an acoustic resonator with a magnetic bearing (aRMB) that overcomes the long‐standing size‐performance trade‐off in low‐frequency sound absorption. At its core, this approach draws upon the fundamental physical principle of negative stiffness, a condition where the potential energy landscape exhibits a local negative curvature, enabling a force that acts in the direction of displacement. By incorporating a ball‐bearing‐guided magnetic ring configuration, the aRMB transforms inherent global instability into a controllable, single‐degree‐of‐freedom negative stiffness. This macroscopic potential landscape, analogous to the inflection point of a potential well, allows weak sound pressure to readily excite the system, thereby facilitating efficient acoustic energy absorption and dissipation through intense energy localization at deep subwavelength scales.

Experimental results demonstrate that the aRMB achieves a stiffness ratio of −0.40 using a cavity depth of 70 mm, marking the first experimental realization of effective magnetic negative stiffness. This configuration yields an absorption bandwidth 65% broader than that of the classic mass‐tuning design. Theoretical analysis further reveals the technology's substantial potential: optimizing structural parameters (reducing the air gap to 2.5 mm and adopting a lightweight conical diaphragm) allows the stiffness ratio to reach ‐0.80. Under these conditions, the total sound absorption over the 31–125 Hz range increases by a factor of 3.21 compared to the best classic design (thermodynamics limit), while the effective bandwidth becomes five times broader.

This study confirms that intelligent mechanical design, rather than volumetric scaling, achieves superior low‐frequency acoustic performance in compact configurations. The aRMB framework effectively decouples acoustic performance from physical dimensions, establishing a foundational platform for next‐generation compact acoustic solutions. Future work will focus on further increasing the negative stiffness ratio and optimizing structural integration for real‐world applications in aerospace, transportation, and consumer electronics, where industry standards critically demand miniaturization and low‐frequency performance.

## Author Contributions

**Zhe Zhang**: formal analysis, writing – review and editing. **Ying Hu**: conceptualization, methodology, investigation, data curation, formal analysis, software, visualization, writing – original draft, validation. **Bohua Huang**: visualization, writing – review and editing. **Hallam Bastin Kilcoyne**: writing – review and editing. **Lixi Huang**: conceptualization, supervision, funding acquisition, writing – review and editing, resources. **Xue Han**: investigation, writing – review and editing. **Ying Hu**: conceptualization, methodology, investigation, data curation, formal analysis, software, visualization, writing – original draft, validation. **Zhe Zhang**: formal analysis, writing – review and editing. **Bohua Huang**: visualization, writing – review and editing. **Xue Han**: investigation, writing – review and editing. **Hallam Bastin Kilcoyne**: writing – review and editing. **Lixi Huang**: conceptualization, supervision, funding acquisition, writing – review and editing, resources.

## Conflicts of Interest

The authors declare no conflicts of interest.

## Supporting information




**Supporting File**: advs76584‐sup‐0001‐SuppMat.docx.

## Data Availability

The data that support the findings of this study are available from the corresponding author upon reasonable request.
