# Supplementary material for: Absorption of Broadband Low‐Frequency Sound Beyond the Thermodynamics Limit: An Acoustic Resonator With Magnetic Bearing
Source: Adv Sci (Weinh). 2026 Jul 16;13(56):e76584. doi: 10.1002/advs.76584 (PMC13373766; doi:10.1002/advs.76584)
Supplement: Supplementary file 1 [file ADVS-13-e76584-s001.docx]

Supporting Information

Title: Absorption of broadband low-frequency sound beyond the thermodynamics limit: an acoustic resonator with magnetic bearing

Ying Hu, Zhe Zhang, Bohua Huang, Xue Han, Hallam Bastin Kilcoyne, and Lixi Huang*

Appendix A: Details for absorption coefficient fitting

Before using the measured sound absorption coefficients ($\alpha$) for fitting, an effective fitting frequency range needs to be selected. According to Equation (9), $\alpha^{-1}$ varies linearly with respect to $\left( f_{1}-f_{1}^{-1} \right)^{2}$, where $f_{1}=f/f_{res}$ is frequency normalized by the resonance frequency $f_{\mathrm{res}}$,

$$\alpha^{-1}=\frac{\left( 1+D \right)^{2}}{4D}+\frac{\Gamma^{2}}{4D}\left( f_{1}-f_{1}^{-1} \right)^{2}$$

(S1)

This linearity criterion offers a means to both assess the impedance model's validity and determine the effective data range. Taking the test data for aRMB under the condition of a 70 mm cavity with type 3 magnets as an example, the results are shown in **Figure S1**. The linear fitting result shows that the data within the frequency range of $\alpha\geq0.3\alpha_{max}$ are suitable for fitting. Data quality may suffer from two factors: measurement errors arising in the low frequency region where the microphone separation is inadequate for long waves, and at high frequencies due to non-piston behavior.

**Figure S1**. Linear fitting for aRMB under the condition of a 70 mm cavity with type 3 magnets.

When $f=f_{res}$, reactance vanishes, $\mathrm{Im}\left\{ Z_{int} \right\}=0$, and the normalized impedance $Z_{int}=D.$ Therefore, the complex reflection coefficient at the interface position is

$$\frac{\hat{p}_{ref}}{\hat{p}_{inc}}=\frac{D-1}{D+1},$$

(S2)

If $D>1$, then $\hat{p}_{ref}/\hat{p}_{inc}>0$, and the phase $\theta=0^{\circ}$ (or 0 radians). If $D<1$, then $\hat{p}_{ref}/\hat{p}_{inc}<0$, and the phase $\theta={180}^{\circ}$ (or $\pi$ radians). According to this method, the $D$ values measured in this study are all greater than 1.

Appendix B: Eigenmodes analysis

An acrylic thin plate with a thickness of 2 mm is used as a vibrating plate and is sealed by a rubber ring to ensure uniform piston-like movement. The soft rubber ring allows the vibrating system to oscillate freely in the axial direction and introduces an insignificant axial restoring force. To verify the feasibility of this design, the eigenmodes of a complete vibrating system (oscillating plate, steel rod, and free magnet) with a fixed constraint at the rubber outer ring were analyzed using solid module simulation in COMSOL.

As shown in **Figure S2 (a-c)**, the results display that the first eigenmode occurs at about 18.2 Hz. In this first mode, the vibration system performs a nearly piston-like motion. Due to the inevitable positive stiffness of the structure, it is difficult to have a first-order mode near zero frequency. However, it is low enough relative to the source frequency range used in the experiments (40-200 Hz). The second-order mode (*f*_(2)_ = 532.3Hz) and third-order mode (*f*_(3)_ = 982.6Hz) are far away from the sound source frequency range, which further proves the feasibility of the design.

Replacing the acrylic plate with a conical diaphragm can not only provide sufficient overall rigidity but also reduce the weight. Through modal analysis calculations, the strategy of using conical diaphragm can achieve similar mode characteristics while reducing the mass by one third, as shown in **Figure S2 (d-f)**.

**Figure S2**. (a-c) First three eigenmodes and eigenfrequencies of the complete system in test (rubber-spider ring, oscillating plate, steel rod and free magnet). (d-f) First three eigenmodes and eigenfrequencies of the complete system using a conical diaphragm instead of the oscillating plate*.*

Appendix C: 3D acoustic-solid coupling model

**Figure S3.** 3D simulation result of sound absorption coefficient by a resonator with magnetic negative stiffness with a cavity of 90mm in depth.

The 3D finite element model of the resonator with magnetic negative stiffness was established using the acoustic-solid coupling module in COMSOL. According to the calculation of the magnetic field in Section 2.2, when the moving part oscillates near the original position ($x$ = 0), the magnetic force is approximately 0, and the magnetic negative stiffness reaches its maximum value (see **Figure 5**a-b). In the acoustic-solid coupling model, the magnetic stiffness obtained from the magnetic bearing calculation is directly modeled as a negative virtual spring with a constant stiffness coefficient. Because the instability of the magnet is constrained by mechanical contact, the simulation model can be simplified in this way without requiring a coupled magnetic field module.

The calculation results are shown in **Figure S3**. The absorption curves of the designed resonator under different negative stiffness values are obtained, which are in agreement with the experimental results. The specific parameters used in the three-dimensional acoustic-solid coupling model simulation are shown in **Table S1**.

**Table S1.** The specific parameters used in the 3D acoustic-solid coupling model in COMSOL.

| Parameter | value | Parameter | value |
| --- | --- | --- | --- |
| Cavity depth *L*_c_ | 90×10^-3^ m | Rubber-spider ring thickness | 0.8×10^-3^ m |
| Cavity/duct area $A_{d}$ | 0.102^2^ m^2^ | Rubber-spider ring width | 9.5×10^-3^ m |
| Vibrating plate radius | 36×10^-3^ m | Steel rod radius | 2.5×10^-3^ m |
| Vibrating plate thickness | 2×10^-3^ m | Steel rod length | 24×10^-3^ m |

The negative stiffness applied to the moving body in the simulation is -2000 N/m. According to the simulation results, the total stiffness decreased from an initial value of 21093 N/m to 12808 N/m. The amplification factor is calculated, $\tau=\left( {A_{d}}/{A_{p}} \right)^{2}=4.14$, which leads to an effective oscillating area $A_{p}$ of 0.0051 m².

Appendix D: Theoretical analysis of magnets

Magnets generally have two magnetic poles: the N pole and the S pole. The magnetic field generated by the magnet is directed from the N pole to the S pole. The face-to-face ring magnets and the bearing ring magnets are two fundamental magnetic structures that can generate magnetic negative stiffness. The former utilizes the attractive force between magnets, while the latter utilizes the repulsive force between magnets. Next, the two magnetic structures are compared.

**Figure S4.** (a) Two-dimensional axisymmetric plane diagram of magnetic face-to-face structure and (b) bearing structure.

The face-to-face magnets and the bearing magnets can be seen as solids of revolution, which are obtained by rotating the plane shown in **Figure S4** about the axis OO’. The face-to-face configuration consists of an upper ring magnet and a lower ring magnet, both axially magnetized, as depicted in **Figure S4**(a). The attractive force between the two magnets increases as the air gap decreases, resulting in negative stiffness. Obviously, there is no equilibrium condition in this structure if there are no external constraints. On the other hand, the bearing design comprises an inner ring magnet and an outer ring magnet, also axially magnetized, as shown in **Figure S4**(b). Initially, the magnets are in force equilibrium when aligned horizontally. Once a slight axial movement occurs, the interaction between the same polarity of the upper surfaces and lower surfaces of the inner and outer magnets produces a repulsive force, resulting in a significant negative stiffness effect.

Let positive *x* be pointing upwards, and positive *y* be pointing to the right. Let the center of surface 1 be at the origin, and the center of surface 3 be at $\left( x_{c},y_{c},z_{c} \right)$. Assume surface 1 is within the *yz*-plane, while surface 3 forms an angle of *θ* with the *yz*-plane. Let *R_A,_*_1_, *R_A,_*_2_ be the inner and outer radii of surface 1, and *R_B,_*_1_, *R_B,_*_2_ be the inner and outer radii of surface 3, respectively. The force exerted on surface 3 by surface 1 is written in its x, y, z components as [47]

$$F_{x}(x_{c},y_{c},z_{c})=\pm\frac{\sigma_{s1}\sigma_{s3}}{4\pi\mu_{0}}\iiint\int\frac{\left( x_{c}+r_{3}\cos\beta\sin\theta\right)r_{1}r_{3}d\alpha dr_{1}d\beta dr_{3}}{r^{3}}$$

(S3)

$$F_{y}(x_{c},y_{c},z_{c})=\pm\frac{\sigma_{s1}\sigma_{s3}}{4\pi\mu_{0}}\iiint\int\frac{\left( y_{c}+r_{3}\cos\beta\cos\theta-r_{1}\cos\alpha\right)r_{1}r_{3}d\alpha dr_{1}d\beta dr_{3}}{r^{3}}$$

(S4)

$$F_{z}(x_{c},y_{c},z_{c})=\pm\frac{\sigma_{s1}\sigma_{s3}}{4\pi\mu_{0}}\iiint\int\frac{\left( z_{c}+r_{3}\sin\beta-r_{1}\sin\alpha\right)r_{1}r_{3}d\alpha dr_{1}d\beta dr_{3}}{r^{3}}$$

(S5)

where $\sigma_{Si}=\mu_{0}M_{i}$, $\mu_{0}$ is the permeability of free space, $\mu_{0}=4\pi\times{10}^{-7}$ N/A^2^, and $M_{i}$ is the magnitude of the magnetization in the direction of the outward normal for surface *i*, $M_{i}=900 kA/m$. $r_{1}\in\left[ R_{A,1}, R_{A,2} \right]$, $r_{3}\in\left[ R_{B,1}, R_{B,2} \right]$, $\alpha,\beta\in\left[ 0,2\pi\right]$, and

$$r=\left[ \left( x_{c}+r_{3}\cos\beta\sin\theta\right)^{2}+\left( y_{c}+r_{3}\cos\beta\cos\theta-r_{1}\cos\alpha\right)^{2}+\left( z_{c}+r_{3}\sin\beta-r_{1}\cos\alpha\right)^{2} \right]^{1/2}$$

(S6)

For both structures, the inner radius, outer radius, and thickness of the fixed magnet are *R_A,_*_1_, *R_A,_*_2_ and *h_A_*. The inner radius, outer radius, and thickness of the moving magnet are *R_B,_*_1_, *R_B,_*_2_ and *h_B_*. The displacement of the center of the moving magnet relative to the outer magnet is ∆*x*. Take the counterclockwise angle and moment about an axis to be positive. Let $F=\left[ F_{x} F_{y} F_{z} \right]^{T}$ be the force exerted on the moving magnet by the fixed magnet, then the force exerted on the fixed magnet by the moving magnet is $-F$. Let the geometric center of the fixed magnet be at the origin (0, 0, 0), and that of the moving magnet be at (*x*, *y*, *z*). Let *θ* be the angle of rotation of the moving magnet about the *z*-axis. The overall force between the two ring magnets comes from the interaction forces between different pairs of surfaces:

$$F_{i}=F_{i23}\left( x-\frac{h_{A}}{2}-\frac{h_{B}}{2}\cos\theta,y+\frac{h_{B}}{2}\sin\theta,z \right)+F_{i14}\left( x+\frac{h_{A}}{2}+\frac{h_{B}}{2}\cos\theta,y-\frac{h_{B}}{2}\sin\theta,z \right)+F_{i13}\left( x+\frac{h_{A}}{2}-\frac{h_{B}}{2}\cos\theta,y+\frac{h_{B}}{2}\sin\theta,z \right)+F_{i24}\left( x-\frac{h_{A}}{2}+\frac{h_{B}}{2}\cos\theta,y-\frac{h_{B}}{2}\sin\theta,z \right), i=x,y,z$$

(S7)

Axial stiffness, i.e., x-direction stiffness, is an important performance index that requires attention. Taking the derivative of Equation (S3) with respect to $x_{c}$, the force derivative due to a pair of surfaces given by:

$$\frac{dF_{x}}{dx_{c}}=\pm\frac{\sigma_{s1}\sigma_{s3}}{4\pi\mu_{0}}\int_{R_{B,1}}^{R_{B,2}} \int_{0}^{2\pi} \int_{R_{A,1}}^{R_{A,2}} \int_{0}^{2\pi} \frac{r_{1}r_{3}\left[ r^{2}-3\left( x_{c}+r_{3}\cos\beta\sin\theta\right) \right]d\alpha dr_{1}d\beta dr_{3}}{r^{5}}$$

(S8)

To obtain the overall force derivative, the contributions from different pairs of surfaces are considered:

$$\frac{dF_{x}}{dx}=df_{23}\left( x-\frac{h_{A}}{2}-\frac{h_{B}}{2}\cos\theta,y+\frac{h_{B}}{2}\sin\theta,z \right)+df_{24}\left( x+\frac{h_{A}}{2}+\frac{h_{B}}{2}\cos\theta,y-\frac{h_{B}}{2}\sin\theta,z \right)+df_{13}\left( x+\frac{h_{A}}{2}-\frac{h_{B}}{2}\cos\theta,y+\frac{h_{B}}{2}\sin\theta,z \right)+df_{24}\left( x-\frac{h_{A}}{2}+\frac{h_{B}}{2}\cos\theta,y-\frac{h_{B}}{2}\sin\theta,z \right)$$

(S9)

where$df={{dF}_{x}}/{dx_{c}}.$

Referring to **Figure S5**, suppose ***f*** is the force exerted on a magnetic charge on surface *i* due to another magnetic charge elsewhere, ***f*** is then parallel to the vector connecting the two charges. Let $\boldsymbol{r}_{\boldsymbol{c}}$ denote the vector from the geometric center to the point of action of $\boldsymbol{f}$, $\boldsymbol{r}_{\boldsymbol{v}}$ denote the vector from the geometric center to the center of surface *i*, $\boldsymbol{r}_{\boldsymbol{h}}$ denote the vector from the center of surface *i* to the point of action of ***f***, i.e. $\boldsymbol{r}_{\boldsymbol{h}}\boldsymbol{=}\boldsymbol{r}_{\boldsymbol{c}}\boldsymbol{-}\boldsymbol{r}_{\boldsymbol{v}}$.

**Figure S5.** Torque due to magnetic force between a pair of charges.

For two surfaces 1 and 3, the total torque $T$ about the geometric center is then given by:

$$T=\iint_{S_{1}} \frac{\sigma_{s1}}{S_{3}}\frac{\sigma_{s3}}{4\pi\mu_{0}r^{3}}\left( \boldsymbol{r}_{\boldsymbol{v}}\times\boldsymbol{r}+\boldsymbol{r}_{\boldsymbol{v}}\times\boldsymbol{r} \right)dS_{1}dS_{3}=\boldsymbol{r}_{\boldsymbol{v}}\times\boldsymbol{F}_{\mathbf{13}}+\frac{\sigma_{s1}\sigma_{s3}}{4\pi\mu_{0}}\int_{R_{B,1}}^{R_{B,2}} \int_{0}^{2\pi} \int_{R_{A,1}}^{2\pi} \int_{0}^{R_{A,2}} \frac{2\pi}{r_{1}r_{3}\left( \boldsymbol{r}_{\boldsymbol{h}}\times\boldsymbol{r} \right)d\alpha dr_{1}d\beta dr_{3}}$$

(S10)

$$T_{z}=T_{z1}+T_{z2}$$

(S11)

$$T_{z1}=c_{x}F_{y13}-c_{y}F_{x13}$$

(S12)

$$T_{z2}=\frac{\sigma_{s1}\sigma_{s3}}{4\pi\mu_{0}}*$$

$$\int_{R_{B,1}}^{R_{B,2}} \int_{0}^{2\pi} \int_{R_{A,1}}^{R_{A,2}} \int_{0}^{2\pi} \frac{r_{3}\cos\beta\left( x_{c}\cos\theta-y_{c}\sin\theta+r_{1}\cos\alpha\sin\theta\right)r_{1}r_{3}}{r^{3}}d\alpha dr_{1}d\beta dr_{3}$$

(S13)

where $\left( x_{c},y_{c},z_{c} \right)$ represents the position of the surface 3 center relative to the surface 1 center. $T_{z}$ is the component of the total torque along z-direction, $T_{z1}$ is the torque component due to the total magnetic force $\boldsymbol{F}_{\mathbf{13}}$, $T_{z2}$ is the additional torque component arising directly from the distributed magnetic charges. When $\theta$ = 0, the torque of rotation is also equal to 0 due to the symmetry of the magnetic field.

Based on the above analytical formulas, the magnetic force, torque, and stiffness of the bearing structure are calculated respectively. Additionally, the finite element method (FEM) is adopted to display the magnetic field distribution and magnetic induction lines intuitively.

**Figure S6.** Comparison of axial force and stiffness characteristics between face-to-face magnet structures and bearing magnet structures under the same volume and air gap.

The comparison of the performance of the two types of magnetic structures is illustrated in **Figure S6**. The axial forces and stiffness of magnets with the same total volume and a 1 mm air gap were analyzed. The variable *Q* represents the volume ratio of the moving magnet to the fixed magnet in each structure. For the face-to-face magnet structure, *R*_B,1_=*R*_A,1_=2mm, $h_{B}=h_{A}=3$ mm, and for the bearing magnet, *R*_B,1_=2 mm, $h_{B}=h_{A}=3$ mm. The results indicate that when $Q=1$ (equal volumes for both magnetic structures), the face-to-face magnets exhibit the highest negative stiffness and maximum unbalanced magnetic force on one side. The bearing magnet achieves its peak negative stiffness at $Q=1.6$, reaching approximately 1.96 times that of the face-to-face magnets. Importantly, the bearing magnets have the characteristics of force balance, which helps to simplify the design and reduce tension risks. Consequently, the bearing ring magnets were chosen as the mechanical negative stiffness mechanism for this study. Subsequently, the characteristics of the bearing magnet will be analyzed through the magnetic flux density distribution map, and the stability of the axial, lateral, and rotational degrees of freedom will be further explored.

**Table S2.** The specific dimensions of the three types of bearing magnets.

| Ring magnets | Types | Inside radius (mm) × external radius (mm) × thickness (mm) |
| --- | --- | --- |
| Inner magnet (moving) | Type 1-3 | 2×9×3 |
| Outer magnet (fixed) | Type 1 | 15×20×3 |
|  | Type 2 | 13.5×20×3 |
|  | Type 3 | 12.5×20×3 |

**Figure S7** shows the two-dimensional axisymmetric magnetic field distribution of the bearing structure for three different types obtained by FEM, where y=0 is the axis of rotation. The specific dimensions of the three types of bearing magnets are listed in **Table S2**. Magnetic lines are closed curves that do not actually exist, but we can judge the properties of magnetic fields based on the characteristics of these imaginary magnetic lines. The magnetic induction lines start from the upper surface (N pole) and end at the lower surface (S pole). According to the magnetic induction lines, the two ring magnets are axially magnetized in the same direction of magnetization. The density of the magnetic induction lines indicates the strength of the magnetic field, and the denser the magnetic induction lines, the stronger the magnetic field strength and the greater the magnetic force. The end face of the ring magnet shows the strongest magnetism. The magnetic field distribution of the inner magnet at *x*=-5, *x*= 0 and *x*=5 mm was simulated with a fixed outer ring magnet and a moving inner ring magnet. When *x*=0, because the magnetic induction lines cannot intersect, there will be no magnetic field interaction between the inner and outer ring magnets, so they are in equilibrium. When *x*=-5, the repulsive force between N-N and S-S exceeds the attractive force between N-S, and the tendency of the inner magnetic ring to be pushed down by the magnetic field lines can be intuitively observed. Similarly, at *x*=5, the inner magnetic ring is pushed upward by the repulsive magnetic force. The air gaps between the inner magnet and the outer magnet of type 1-3 are 6 mm, 4.5 mm and 3.5 mm, respectively. Comparing the three types of magnetic bearing structures, with a smaller gap (the larger the inner radius of the outer ring magnet), the magnetic induction line density also increases, which means that the magnetic field strength is enhanced. Therefore, reducing the air gap is the main method to increase the negative magnetic stiffness in this study.

**Figure S7.** Magnetic flux density distribution of two-dimensional axisymmetric ring magnets under the condition of the moving inner magnet at $x=-5$,$x= 0$ and $x=5$ mm with three different types.

Appendix E: Experimental and theoretical characterization of SPL-dependent stability

Due to experimental limitations, the maximum incident sound pressure level (SPL) we can generate is 105 dB. To assess the performance under higher SPLs, we developed a three-dimensional finite element model that fully incorporates the nonlinear magnetic force and the acoustic–structure coupling. Because of the model’s complexity, the magnetic bearing module was first simulated separately to obtain the nonlinear curve of axial negative stiffness versus vibration displacement, shown in **Figure S8** (corresponding to the Type 3 curve in **Figure 3(b)**). Then, the expression for negative stiffness is obtained through fitting, which serves as the stiffness input for the 3D acoustic-solid coupling model.

**Figure S8.** Theoretical calculation results of the axial negative stiffness of the magnetic bearing (type 3).

**Figure S9**. Feasibility verification of the simulation model. (a) Experimental result and (b) simulation result of the absorption coefficients at different incident SPLs of 95 dB, 100 dB, and 105 dB.

To validate the reliability of the simulation model, we further carried out absorption coefficient measurements at SPLs from 95 dB to 105 dB. **Figure S9(a)** presents the experimentally measured absorption coefficients at incident SPLs of 95, 100, and 105 dB, while **Figure S9(b)** shows the corresponding simulation results. In the experiment, the measured absorption curves from 95 dB to 105 dB are nearly identical. The stiffness and mass remain essentially unchanged over this range, and the simulation reproduces these results well, which confirms the validity of the model. Meanwhile, the damping also exhibits no measurable variation. This constant damping behaviour arises because the vibration displacement amplitude is only a few microns to a few tens of microns. At such microscopic oscillations, the bearing friction is dominated by viscous shear within the lubricant film rather than Coulomb dry friction; the bearing therefore behaves as a nearly linear viscous damper with a constant damping coefficient. Consequently, the bearing‑induced damping does not introduce measurable amplitude dependence. These experimental and numerical results together demonstrate that the designed aRMB maintains stable absorption performance under typical high‑intensity noise conditions (e.g., industrial noise, engine near‑fields). It is worth noting that the comparative experiments presented in the article were all conducted at an SPL of 95 dB.

**Figure S10.** Simulation result of axial displacement amplitudes at higher incident SPLs.

**Figure S11.** Simulation result of absorption coefficient at higher incident SPLs, 110 to130 dB.

**Figure S10** displays the vibration amplitudes at different incident SPLs obtained from the simulation, and **Figure S11** gives the corresponding absorption coefficient spectra at higher incident SPLs of 110 dB to 130 dB. As shown in **Figure S10(a–c)**, the vibration amplitude remains within the axial effective working range (i.e., the negative‑stiffness range, $x_{0}\approx\pm3.35$ mm) for incident SPLs up to 127 dB, and although the absorption curve progressively exhibits more pronounced nonlinear deviations from the ideal response as the SPL increases (see **Figure S11**), the resonator remains stable and fully functional. When the incident SPL exceeds 127 dB, the vibration amplitude moves beyond the working range $x_{0}$ (see Figure S10(d)), where the magnetic stiffness becomes positive. As a result, the absorption performance degrades first at higher frequencies (see dotted line in Figure S11). This is because the actual air cavity stiffness $k_{\text{air}}(f)$ is lower at higher frequencies due to accumulated phase in waves, making the effective negative stiffness ratio $\kappa(f)$ substantially more negative there. Consequently, the high‑frequency portion of the absorption band operates with a narrower stability margin and is more sensitive to the nonlinear hardening of the magnetic stiffness, leading to an earlier breakdown of the impedance matching condition. The simulation result confirms that the effective operational SPL limit of the aRMB (type 3) is 127 dB. In future work, we plan to explore modifying the magnetic bearing structure parameter or magnetic arrangement to further widen the negative-stiffness range and raise the maximum tolerable SPL threshold.

Appendix F: Test results for different cavity depths

In order to demonstrate the stability and applicability of the designed aRMB, experiments were conducted at different cavity depths. Here, for the 90 mm, 80 mm, and 60 mm specifications, the sound absorption curves for the three configurations are compared to clearly demonstrate this experimental breakthrough. All open circles are experimental data, while the solid lines are the $\alpha$ fitting for the data by the simple resonator with constant mass, damping, and stiffness. The black curves are the results before the fixed magnets are used, namely the ‘without-magnetic-stiffness’ configuration, while the blue curves are the results when fixed magnets (type 3) are installed. The comparison serves the purpose of illustrating the shift in resonance frequency by reducing the system stiffness alone while the mass remains the same.

In **Figure S12**(a), the resonance frequency is reduced from 79.1 Hz for the ‘without-magnetic-stiffness’ design to 57.8 Hz for the ‘with-magnetic-stiffness’ design. Based on the ‘with-magnetic-stiffness’ configuration for $L_{c}=90$ mm, we now take the cavity stiffness $k_{\mathrm{cav}}=4219$ N/m alone and add a perforated panel to form the classic design such that the same resonance frequency of 57.8 Hz is achieved while the dimensionless overall damping parameter remains the same as the ‘with-magnet’ case, which is $D=2.14$. The panel parameters are determined by solving the real and imaginary impedance equations at resonance using grid search and local optimization, leveraging the monotonic relationship between the reactance ratio $Z_{i}/Z_{r}$ and perforation diameter $a$.

For this 90 mm cavity shown in **Figure S12**(a), the panel in the classic design has the following parameters: $a=3.07 \mathrm{mm}, \sigma=0.14\%, t_{p}=10 \mathrm{mm}$, yielding $D=2.14$, $M=118.3$, and $\kappa=0$ (meaning no structural stiffness $k_{\mathrm{rub}}=0$). When two designs share the same resonance frequency and the same cavity size, their performance comparison can be conducted simply by the quality factor defined as $\gamma=\Delta f/f_{res}$, where $\Delta f$ is the full width at half maximum (FWHM) and $f_{\mathrm{res}}$ is the resonant frequency. For the 90 mm cavity, $\gamma$ is 0.29 for the classic design, compared to 0.45 for aRMB design. Similarly, for an 80 mm cavity in **Figure S12**(b), the values are 0.27 and 0.42, respectively. This trend continues at 60 mm in **Figure S12**(c), with values of 0.25 and 0.39. In summary, the aRMB configurations obviously broaden the low-frequency sound absorption bandwidth and significantly improve the quality factor.

**Figure S12.** Comparisons of sound absorption coefficient curves between the aRMB design in experiments and the classic design of perforated panel covering a pure air cavity at different cavity depths. (a) $L_{c}=90 mm$. Case 0 (test data): without magnetic stiffness. Case 0 (fitting): $D=2.06$, $M=76.9$, $\kappa=0.22$. Case 1 (classic design): $D=2.14$, $M=118.3$, $\kappa=0$. Case 2 (test data): with magnet type 3. Case 2 (fitting): $D=2.14$, $M=76.9$, $\kappa=-0.35$. (b) $L_{c}=80 mm$. Case 0 (test data): without magnetic stiffness. Case 0 (fitting): $D=2.14$, $M=88.4$, $\kappa=0.17$. Case 1 (classic design): $D=2.14$, $M=131.4$, $\kappa=0$. Case 2 (test data): with magnet type 3. Case 2 (fitting): $D=2.14$, $M=84.7$, $\kappa=-0.35$. (c) $L_{c}=60 mm$. Case 0 (test data): without magnetic stiffness. Case 0 (fitting): $D=2.25$, $M=118.7$, $\kappa=0.11$. Case 1 (classic design): $D=2.38$, $M=185.4$, $\kappa=0$. Case 2 (test data): with magnet type 3. Case 2 (fitting): $D=2.38$, $M=76.9$, $\kappa=-0.36$.

Appendix G: Simulated performance and nonlinear stability of the advanced design

To better evaluate the rationality of the parameters of the advanced design with conical membrane and air gap of 2.5 mm, a three-dimensional simulation model was established. The axial negative-stiffness data points for an air gap of 2.5 mm were obtained through theoretical calculation of the magnetic bearing, as shown by the open blue squares in **Figure S13**. The expression of axial negative stiffness as a function of displacement was then extracted by fitting, yielding an effective working range of $x_{0}\approx\pm2.92$ mm.

**Figure S13.** Theoretical calculation results of the axial negative stiffness of the magnetic bearing with the air gap of 2.5 mm.

**Figure S14.** Simulation result of axial displacement amplitudes at higher incident SPLs of the advanced design with conical membrane structure and air gap of 2.5 mm.

**Figure S14** presents the displacement amplitude response of the conical diaphragm structure at different incident SPLs, and **Figure S15** shows the corresponding absorption coefficient spectra. As the SPL increases, the vibration amplitude grows and the absorption curve exhibits a progressively more pronounced shift relative to the ideal constant‑stiffness curve. This shift arises from the inherent nonlinearity of the magnetic stiffness: at larger displacements, the operating point moves into the nonlinear region of the stiffness–displacement curve, causing the effective stiffness to deviate from its low‑amplitude value and thereby altering the resonance condition and the absorption profile. Nevertheless, up to 122 dB the vibration amplitude remains within the effective working range, and the system continues to operate stably. Beyond 122 dB, the vibration amplitude exceeds the effective working range $x_{0}$ and the magnetic stiffness becomes positive. This failure initiates at higher frequencies, where the air stiffness is lower due to the effect of accumulated wave phase, making the impedance matching condition more sensitive to the nonlinear hardening of the magnetic stiffness, and thereby establishing 122 dB as the effective operational threshold of this design. This result provides theoretical confirmation that the advanced design can sustain stable absorption performance under a reasonably high-intensity noise condition.

**Figure S15.** Simulated absorption coefficient of the advanced design at different incident SPLs.
